# Medium Entropy‐Enabled High Performance Cubic GeTe Thermoelectrics

**DOI:** 10.1002/advs.202100220

**Published:** 2021-05-06

**Authors:** Shizhen Zhi, Jibiao Li, Lipeng Hu, Junqin Li, Ning Li, Haijun Wu, Fusheng Liu, Chaohua Zhang, Weiqin Ao, Heping Xie, Xinbing Zhao, Stephen John Pennycook, Tiejun Zhu

**Affiliations:** ^1^ College of Materials Science and Engineering Shenzhen Key Laboratory of Special Functional Materials Guangdong Research Center for Interfacial Engineering of Functional Materials Guangdong Provincial Key Laboratory of Deep Earth Sciences and Geothermal Energy Exploitation and Utilization Institute of Deep Earth Sciences and Green Energy Shenzhen University Shenzhen 518060 China; ^2^ Center for Materials and Energy (CME) and Chongqing Key Laboratory of Extraordinary Bond Engineering and Advanced Materials Technology (EBEAM) Yangtze Normal University Chongqing 408100 China; ^3^ Institute for Clean Energy and Advanced Materials Southwest University Chongqing 400715 China; ^4^ Department of Materials Science and Engineering National University of Singapore Singapore 117575 Singapore; ^5^ State Key Laboratory for Mechanical Behavior of Materials Xi'an Jiaotong University Xi'an 710049 China; ^6^ State Key Laboratory of Silicon Materials and School of Materials Science and Engineering Zhejiang University Hangzhou 310027 China

**Keywords:** band engineering, entropy engineering, GeTe, phase transition, thermoelectric

## Abstract

The configurational entropy is an emerging descriptor in the functional materials genome. In thermoelectric materials, the configurational entropy helps tune the delicate trade‐off between carrier mobility and lattice thermal conductivity, as well as the structural phase transition, if any. Taking GeTe as an example, low‐entropy GeTe generally have high carrier mobility and distinguished *zT* > 2, but the rhombohedral‐cubic phase transition restricts the applications. In contrast, despite cubic structure and ultralow lattice thermal conductivity, the degraded carrier mobility leads to a low *zT* in high‐entropy GeTe. Herein, medium‐entropy alloying is implemented to suppress the phase transition and achieve the cubic GeTe with ultralow lattice thermal conductivity yet decent carrier mobility. In addition, co‐alloying of (Mn, Pb, Sb, Cd) facilitates multivalence bands convergence and band flattening, thereby yielding good Seebeck coefficients and compensating for decreased carrier mobility. For the first time, a state‐of‐the‐art *zT* of 2.1 at 873 K and average *zT*_ave_ of 1.3 between 300 and 873 K are attained in cubic phased Ge_0.63_Mn_0.15_Pb_0.1_Sb_0.06_Cd_0.06_Te. Moreover, a record‐high Vickers hardness of 270 is attained. These results not only promote GeTe materials for practical applications, but also present a breakthrough in the burgeoning field of entropy engineering.

## Graphical abstract

The power of medium‐entropy thermoelectrics is exploited in GeTe by co‐alloying Mn, Pb, Sb, and Cd to optimize the delicate trade‐offs between the carrier mobility, crystal structure, and lattice thermal conductivity. For the first time, a distinguished peak *zT* ≈ 2.1 and high Vickers hardness *H*_V_ ≈ 270 are attained in the cubic Ge_0.63_Mn_0.15_Pb_0.1_Sb_0.06_Cd_0.06_Te with no phase transition.

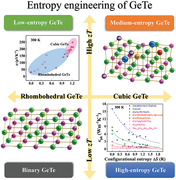

## 1 Introduction

More than two‐thirds of the energy produced worldwide is wasted in the form of heat. Thermoelectrics recovers waste heat through direct heat‐to‐electrical energy conversion, leaving minimal environmental footprint.^[^ ^1^, ^2^ ^]^ The conversion efficiency of a thermoelectric (TE) device is governed by the materials’ figure of merit, *zT* = *σα* ^2^ *T*/*κ*, where *T*, *σ*, *α*, and *κ* are the absolute temperature, electrical conductivity, Seebeck coefficient, and thermal conductivity (including the carrier component *κ*_el_ and the phonon component *κ*_ph_), respectively. These TE properties are interdependent via the carrier concentration *n*_H_. Band engineering^[^ ^3^, ^4^ ^]^ and phonon engineering^[^ ^5^ ^]^ have been two mainstream strategies for enhancing *zT*. Quite some TE materials undergo phase transition with varying temperature, manipulating crystal lattice symmetry through phase engineering can extend the thermoelectrically favorable phase and suppress the undesired one.^[^ ^6^, ^7^, ^8^, ^9^ ^]^

Notably, entropy engineering has emerged as a paradigm‐shifting strategy that integrates the phase, phonon, and band engineering via multi‐principal‐element alloying.^[^ ^10^, ^11^, ^12^, ^13^, ^14^ ^]^ The configurational entropy Δ*S* is an effective descriptor in the materials genome approach to developing high performance TE materials. Specially, the trade‐off between the carrier mobility *μ*_H_ and the *κ*_ph_, largely governed by Δ*S*, is outstanding and delicate.^[^ ^1^ ^]^ For traditional low‐entropy alloys (LEAs) with Δ*S* < 1*R*,^[^ ^15^ ^]^ where *R* is the gas constant, doping is the routine to increase the *zT* by simultaneously optimizing the *n*_H_ and scattering high‐frequency phonons.^[^ ^16^ ^]^ Thus, LEAs generally have both high *μ*_H_ and *κ*_ph_, limiting the *zT* within a range of 1–2.^[^ ^10^ ^]^ In addition, doping in LEAs is inherently restricted by the low solubility limit of dopants, thereby restricting the phase space for performance optimization.^[^ ^10^, ^14^ ^]^

High‐entropy alloys (HEAs) have attracted increasing interest in TE community.^[^ ^10^, ^11^, ^12^, ^13^, ^14^ ^]^ The basic concept is alloying five or more principal elements, each with the atomic percentage of between 5% and 35% to attain high Δ*S* > 1.5*R*.^[^ ^15^ ^]^ Importantly, the core effects of HEAs are closely associated with the transport properties. i) High‐entropy effect can attain the high‐symmetry crystal structure that are favorable for high band degeneracy *N*_V_ and hence large *α*.^[^ ^11^ ^]^ ii) The atomic size difference between multiple elements leads to severe lattice‐distortion effect and promotes the formation of multiscale microstructures, effectively scattering wide‐wavelength phonons.^[^ ^10^ ^]^ Although HEAs lower the *κ*_ph_ down to its theoretical minimum value, high entropy itself does not suffice high *zT* because of the concurrently degraded *μ*_H_.^[^ ^10^ ^]^

It is unnecessary to equate HEA to high *zT*. On one hand, LEAs have the advantage of large *μ*_H_, while HEAs possess an ultralow *κ*_ph_, both of these effects may be balanced in medium‐entropy alloys (MEAs) with 1*R* < Δ*S* < 1.5*R*.^[^ ^15^ ^]^ On the other hand, for most TE materials with moderate initial *κ*_ph_ (1–6 W m^–1^ K^–1^) and high (cubic) or intermediate (rhombohedral, hexagonal, tetragonal) crystal symmetry, medium‐entropy alloying is sufficient to decrease the *κ*_ph_ to its glass‐like limit and stabilize the high‐symmetry cubic structure at room temperature.^[^ ^11^ ^]^

Here, GeTe, as a promising Pb‐free substitute for mid‐temperature power generations,^[^ ^17^, ^18^ ^]^ is chosen as a model system for validating the efficacy of medium‐entropy thermoelectrics. As known, binary GeTe undergoes a rhombohedral (R‐GeTe) to cubic (C‐GeTe) phase transition around 700 K.^[^ ^17^, ^18^ ^]^ After extensive research on GeTe LEAs, both low‐temperature R‐GeTe^[^ ^7^, ^19^ ^]^ and high‐temperature C‐GeTe^[^ ^20^, ^21^, ^22^, ^23^, ^24^, ^25^, ^26^, ^27^, ^28^, ^29^, ^30^, ^31^, ^32^, ^33^, ^34^, ^35^, ^36^, ^37^, ^38^ ^]^ show excellent *zT* > 2, which is known as a prerequisite to make TE devices competitive. However, the existing phase transition may damage the GeTe‐based materials or the material/electrode interfaces during usage, limiting their practical applications.^[^ ^39^ ^]^

High‐entropy alloying can depress the phase transition of GeTe. Unfortunately, the drastically reduced *μ*_H_ impedes the realization of high *zT* > 2. For instance, single‐phase cubic structures were stabilized in Ge_0.25_Sn_0.25_Pb_0.25_Mn_0.25_Te (Δ*S* = 1.39 *R*)^[^ ^11^ ^]^ and Ge_1/3_Sn_1/3_Pb_1/3_Te_1/3_Se_1/3_S_1/3_ (Δ*S* = 2.2 *R*),^[^ ^40^ ^]^ while their peak *zT* values were only 0.92 at 800 K and 0.51 at 375 K, respectively. *P*‐type (GeTe)_75_(AgSbSe_2_)_25_^[^ ^41^ ^]^ and (GeTe)_75_(AgBiSe_2_)_25_^[^ ^42^ ^]^ also obtained the ambient cubic structures. But their maximum *zT* values were only 1.5 and 0.1 at 720 K, respectively.

To this end, medium‐entropy alloying may gain the advantages of both low‐entropy (high *zT* > 2) and high‐entropy GeTe (no phase transition). Medium‐entropy alloying has other advantages in GeTe‐based alloys. First, GeTe has the highest *κ* and lowest *μ*_H_ among the *M*Te (*M* = Pb, Sn, Ge),^[^ ^17^, ^18^ ^]^ assuring that medium‐entropy alloying can strongly reduce the *κ*, but produces a moderate impact on the carrier transport. Economically, multi‐principal‐element substitution greatly reduces the content of expensive Ge, saving the cost for mass production. Technically, medium‐entropy GeTe inherently has better mechanical properties relative to low‐entropy GeTe.

It should be emphasized again that the high Δ*S* itself does not ensure high *zT* in light of the declined *μ*_H_.^[^ ^10^ ^]^ Consequently, reasonably selecting the alloying species, to compensate the low *μ*_H_ by band engineering, is a prerequisite for realizing the *zT* improvement. Based on previous studies,^[^ ^24^, ^25^, ^27^, ^43^, ^44^, ^45^ ^]^ alloying Mn, Pb, Sb, and Cd at the Ge site is selected here. First, all these elements have high solid solubility (>5 mol%) in GeTe (Table S1, Supporting Information). In addition, Mn and Cd enable the convergence of multivalence bands,^[^ ^27^, ^43^, ^44^, ^45^ ^]^ while Pb and Sb reduce the too high *n*_H_ of GeTe via increased formation energy of Ge vacancies^[^ ^24^ ^]^ and aliovalent doping,^[^ ^25^ ^]^ respectively. Although sole Mn/Pb/Sb/Cd doping or Mn‐Sb/Cd‐Sb/Pb‐Sb dual‐doping has been reported early,^[^ ^24^, ^25^, ^27^, ^43^ ^]^ there are no reports on (Mn, Pb, Sb, Cd) co‐alloying GeTe, that is, medium‐entropy GeTe. Despite a small number of toxic Pb (10 mol%) and Cd (6 mol%) is adopted in this work, the use of toxic elements is reduced by more or less an order of magnitude compared to PbTe‐based alloys. Further, the toxicity and high *zT* values are a pair of trade‐off as the materials performance is important for thermoelectrics in the first place.

From a phase perspective, adding an extra element in the raw materials is adding a new dimension to the phase space. In this work, entropy engineering is implemented to GeTe by stepwise alloying Mn, Pb, Sb, and Cd to simultaneously achieve pure cubic structure and distinguished *zT* > 2. As a result, a state‐of‐the‐art *zT* ≈ 2.1 at 873 K, high average *zT*_ave_ of 1.3 between 300 and 873 K and record high Vickers hardness *H*_V_ of 270 are concurrently attained in cubic Ge_0.63_Mn_0.15_Pb_0.1_Sb_0.06_Cd_0.06_Te, attesting to the efficacy of “medium‐entropy thermoelectrics.”

## 2 Results and Discussion

### 2.1 Suppressing the Phase Transition by Medium‐Entropy Alloying

Attaining pure C‐GeTe without phase transition is not only crucial for the long‐term reliability of TE devices, but also is conducive to high *zT* over the entire temperature range. The foremost reason is the C‐GeTe has two valence bands (VBs) with a small energy offset (Δ*E*), which however transfer into multi sub‐VBs separated by a large Δ*E* in the R‐GeTe, giving rise to a lower *N*_V_ and *α*.^[^ ^17^, ^18^ ^]^ Thus, it is expected to depress the phase transition through medium‐entropy alloying.

Thermodynamically, raising Δ*S* tends to endow the GeTe‐based alloys with a high‐symmetry cubic structure.^[^ ^11^, ^40^, ^41^, ^42^ ^]^ For a solid solution comprising *n* components, each with mole fractions *x*_i_, Δ*S* is defined as follows:^[^ ^11^, ^15^ ^]^

(1) $$\Delta S=-R\sum_{i=1}^{n}x_{i}\ln x_{i}$$

**Figure 1:**
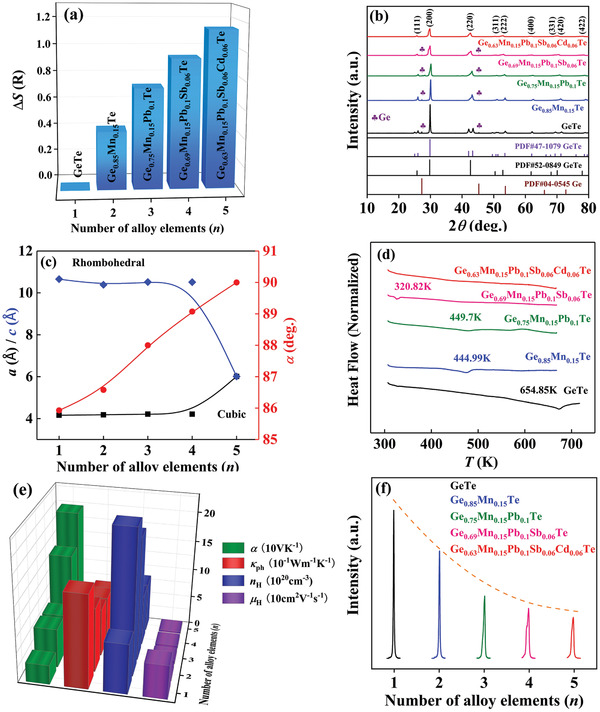
Room temperature a) configurational entropy Δ*S*, b) unnormalized powder XRD patterns, c) lattice parameter *a* and *c* and interaxial angle *α* of GeTe‐based alloys. d) DSC curves and the obtained phase transition temperature *T*_0_ of GeTe‐based alloys. e) Room‐temperature Seebeck coefficient *α*, lattice thermal conductivity *κ*_ph_, carrier concentration *n*_H_, and carrier mobility *μ*_H_ of GeTe‐based alloys. f) The enlarged (200) Bragg peak in unnormalized powder XRD patterns of GeTe‐based alloys.

Predictably, Δ*S* is considerably increased with increasing numbers of alloying elements in our GeTe‐based alloys. For example, the Δ*S* substantially increased from 0 *R* for GeTe to 0.73 *R* for Ge_0.75_Mn_0.15_Pb_0.1_Te, and then increases to 1.14 *R* for Ge_0.63_Mn_0.15_Pb_0.1_Sb_0.06_Cd_0.06_Te, reaching the medium‐entropy region with 1 *R* < Δ*S* < 1.5 *R* (**Figure** **1**a). As seen from the room‐temperature powder X‐ray diffraction (XRD) pattern (Figure 1b), the double peaks in the 2*θ* range of 23°–27° and 41°–45° indicate that pristine GeTe crystallizes in the rhombohedral structure.^[^ ^43^ ^]^ With increasing Δ*S*, the double peaks gradually merge and become a single peak, demonstrating that the room‐temperature structure gradually changes from R‐GeTe to C‐GeTe. With ascending Δ*S*, the increase in both lattice parameter *a* and interaxial angle *α*, while the decrease in lattice parameter *c*, concurrently confirms that the phase transition from R‐GeTe to C‐GeTe is promoted (Figure 1c).^[^ ^33^ ^]^ Moreover, the increasing Δ*S* gradually lengthens the short Ge—Te bond length and shortens the long bond length (Figure S1, Supporting Information), which further confirms this conclusion.

Differential scanning calorimetry (DSC) analysis (Figure 1d and Figure S2, Supporting Information) was implemented to detect the evolution of phase transition temperature *T*_0_ with respect to increasing Δ*S*. The pristine GeTe undergoes a phase transition at the high temperature of ≈655 K. *T*_0_ follows the law of a single dopant in the low‐entropy region: Mn doping reduces *T*_0_,^[^ ^43^ ^]^ while Pb doping has a weak influence on *T*_0_.^[^ ^24^ ^]^ When Δ*S* approaches or surpasses 1*R*, a substantial drop in *T*_0_ is observed. Eventually, the cubic phase stabilizes at 300 K for the Ge_0.63_Mn_0.15_Pb_0.1_Sb_0.06_Cd_0.06_Te sample. Despite sole Mn or Sb doping reduces the *T*_0_ to some extent, Mn‐Sb dual‐doping cannot eliminate the phase transition in previous studies and this work.^[^ ^25^, ^43^ ^]^ The formation of C‐GeTe in this work is attributed to the increased Δ*S*. To cross‐check this conclusion, the low‐temperature DSC measurement from 190 to 673 K was adopted to the Ge_0.63_Mn_0.15_Pb_0.1_Sb_0.06_Cd_0.06_Te sample, indicating the *T*_0_ is even below 190 K (Figure S2f, Supporting Information). This further confirms that medium‐entropy alloying is sufficient to eliminate the phase transition of GeTe‐based alloys.

Tailoring the entropy to an optimal range could maximize the *μ*_H_/*κ*_ph_ ratio for a given material.^[^ ^1^, ^10^ ^]^ As expected, the continuously increasing Δ*S* simultaneously enhances *S* and reduces *κ*_ph_ (Figure 1e). Despite medium‐entropy alloying is favorable for attaining decent *μ*_H_, the room‐temperature *μ*_H_ falls drastically from 90 cm^2^ V^–1^ s^–1^ for pristine GeTe to 7.5 cm^2^ V^–1^ s^–1^ for Ge_0.85_Mn_0.15_Te and then to 3.4 cm^2^ V^–1^ s^–1^ for Ge_0.63_Mn_0.15_Pb_0.1_Sb_0.06_Cd_0.06_Te. The deterioration of *μ*_H_ in this study mainly originates from the band flattening upon Mn alloying and persistently enhanced alloy scattering, which will be discussed later. Another crucial factor is the variations in *n*_H_. Pristine GeTe exhibits a high *n*_H_ of 8.4 × 10^20^ cm^–3^ at 300 K owing to the intrinsic Ge vacancies.^[^ ^33^, ^46^, ^47^ ^]^ Isovalent Mn substitution largely increases the *n*_H_ to 2.4 × 10^21^ cm^–3^, while the Pb replacement slightly reduces the *n*_H_ to 2.1 × 10^21^ cm^–3^, which are related to the formation energy variation of Ge vacancies.^[^ ^24^, ^43^, ^44^ ^]^ Subsequently, the aliovalent Sb^3+^ serves as effective electron donor and decreases the *n*_H_ to 8.7 × 10^20^ cm^–3^. Theoretically, sole Cd doping has no obvious influence on *n*_H_.^[^ ^27^ ^]^ Interestingly, the addition of Cd in this study further reduces *n*_H_ to 8.3 × 10^20^ cm^–3^, which may be due to the higher solubility of Ge in GeTe‐based MEAs due to high‐entropy effects.^[^ ^10^ ^]^ More detailed discussion can be found in Supporting Information and Figures S3–S5, Supporting Information.

### 2.2 Lattice‐Distortion and Multiscale Microstructures

Attaining C‐GeTe without phase transition can accelerate the practical applications of GeTe‐based alloys and obtain large *α* owing to high *N*_V_. In the following section, we aim to implement multiscale microstructures in GeTe‐based MEAs to attain the ultralow *κ*_ph_.

Due to the atomic size difference among the various components, severe lattice distortion can be introduced into our cubic GeTe‐based MEAs.^[^ ^15^ ^]^ As shown in the enlarged view of (200) Bragg peaks for our GeTe‐based alloys (Figure 1f), the gradual decrease in XRD peak intensity with increasing Δ*S* is a clear evidence of intrinsic lattice distortions, resulting from the increased diffuse scattering.^[^ ^15^, ^48^ ^]^ Hence, a high‐symmetry cubic structure and severe distorted lattice are simultaneously achieved in our GeTe‐based MEAs, which are respectively favorable for good electrical power factor PF = *σα* ^2^ and ultralow *κ*_ph_.

**Figure 2:**
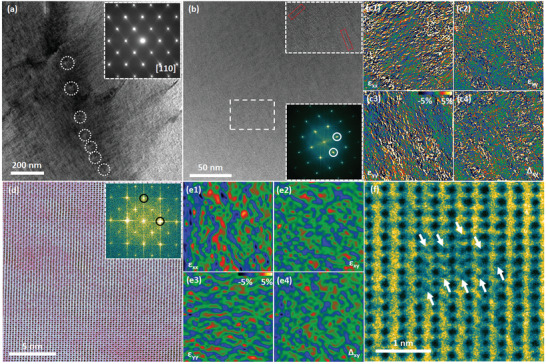
Microstructure of Ge_0.63_Mn_0.15_Pb_0.1_Sb_0.06_Cd_0.06_Te. a) TEM image showing twin‐free and strained structure. These white circles indicate the dislocation arrays. b,c) Electron diffraction patterns along [001] and [110] zone axes. d) HRTEM image, with FFT image inset; the other inset is enlarged image showing two linear defects. e1,e2) Strain analysis of (d) showing high strain. f) Atomically‐resolved STEM ABF image with FFT image inset. g1–g4) Strain analysis of (f). h) Enlarged image showing interstitial cluster.

In order to reveal the structural origin of the severe lattice distortion and its effect on the microstructure, (scanning) transmission electron microscopy (STEM/TEM) was performed on the cubic Ge_0.63_Mn_0.15_Pb_0.1_Sb_0.06_Cd_0.06_Te sample (**Figure** **2**), in which the lowest *κ*_ph_ was achieved. In addition to tiny amounts of micron‐size Mn second phase, the electron probe microanalysis reveals that all elements are homogeneously distributed in the matrix for the (Mn, Pb, Sb, Cd) co‐alloyed sample (Figures S5 and S6, Supporting Information). The TEM image in Figure 2a shows strained microstructures with dislocation arrays and sub‐nanoscale strain clusters due to severe lattice distortion, presenting a high strain contrast in the TEM image. There is no domain variants and twinned structures observed in this sample in light of depressed phase transition, in contrast to the classical microstructures of R‐GeTe.^[^ ^49^ ^]^ Figure 2b,c shows the electron diffraction patterns along the [001] and [110] zone axes, where *a* ≈ 0.6 nm and *α* = 90°, respectively; these patterns are not consistent with R‐GeTe, but with C‐GeTe. Figure 2d is a high‐resolution TEM (HRTEM) image showing a strained lattice due to lattice distortion as well as some linear defects, as the enlarged image shows. These linear defects are ordered Ge vacancies owing to the migration and recombination of Ge vacancies, which are another typical microstructure of R‐GeTe. But the density of ordered Ge vacancies in our C‐GeTe MEAs is much lower than that of corresponding R‐GeTe LEAs.^[^ ^27^, ^34^, ^36^ ^]^

To reveal the internal strain of the lattice distortion, the image was analyzed by geometric phase analysis (GPA)^[^ ^50^ ^]^ that is a semi‐quantitative lattice image‐processing approach for revealing the spatial distribution of relative elastic strain. As shown in Figure 2e1,e2, the entire area is strained along different directions. To further confirm the lattice distortion in the material, aberration‐corrected STEM was employed to acquire an atomically resolved *Z*‐contrast image, by which the local structural information could be resolved in more details. As depicted in Figure 2f, the lattice is not homogeneous but possesses sub‐nanoscale clusters with a darker contrast compared with that of the matrix, owing to the strain contrast. We also performed GPA for the strain analysis. This indicates that the strains due to lattice distortion are distributed widely in the material. Moreover, the strain is anisotropic and therefore observed along different directions, as shown in Figure 2g1–g4. The majority of multiple dopants are distributed as substitutions in the matrix‐possessing internal strains. Some dopants, particularly those with small sizes, for example, Mn, can also squeeze into the lattice of the matrix and become interstitials, similar with those in isotropic PbTe systems.^[^ ^51^, ^52^ ^]^ A cluster of interstitials shown in Figure 2h are most probably Mn atoms that act as the embryo of the micron‐scale Mn second phases (Figures S4 and S5, Supporting Information). The observed nanoscale strain clusters (Figure 2e) could be regarded as strain network, connected by the atomic‐scale substitutions, interstitials, vacancies, and dislocations. Therefore, the medium‐entropy alloying‐induced multiscale microstructures, including atomic‐scale substitutions and Mn interstitials, dislocation arrays, ordered Ge vacancies, sub‐nanoscale strain clusters, and micron‐scale Mn second phases would strongly diminish the *κ*_ph_.

### 2.3 Band Structure Variation upon Medium‐Entropy Alloying

In order to gain deeper insight into the effect of medium‐entropy alloying on the electronic structure of GeTe‐based alloying, we conducted density functional theory calculations for all the samples, as displayed in **Figure** **3** and Figures S7–S20, Supporting Information. As can be seen from Figure 3a,b, R‐GeTe has a larger indirect band gap *E*_g_, whereas C‐GeTe has a smaller direct *E*_g_.

**Figure 3:**
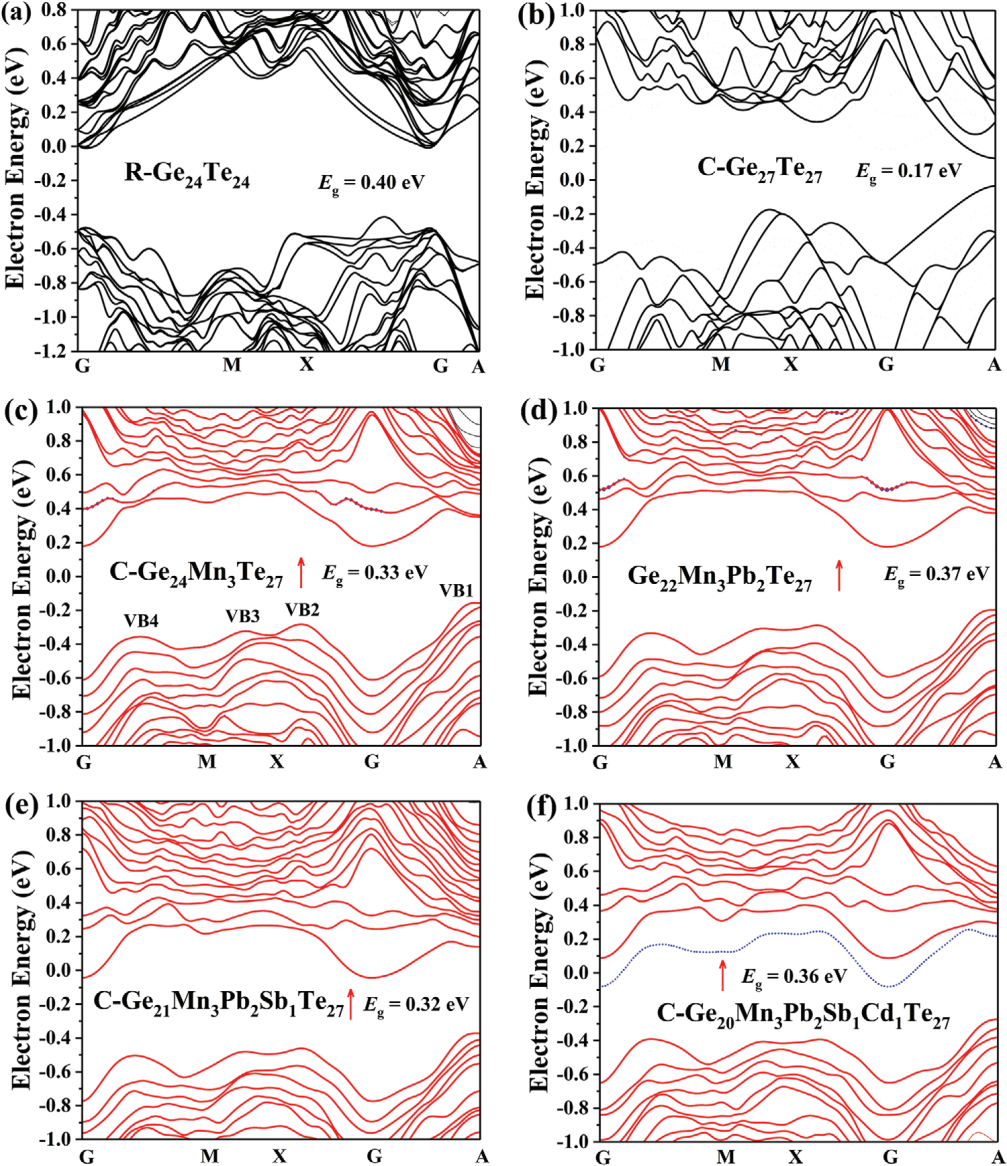
Electron band structures (spin up) of a) R‐Ge_24_Te_24_, b) C‐Ge_27_Te_27_, c) C‐Ge_24_Mn_3_Te_27_, d) C‐Ge_22_Mn_3_Pb_2_Te_27_, e) C‐Ge_21_Mn_3_Pb_2_Sb_1_Te_27_, and f) C‐Ge_20_Mn_3_Pb_2_Sb_1_Cd_1_Te_27_. The Fermi level is positioned at zero energy. The upward red arrow represents the spin up polarized electron band structures.

Figure 3b–f presents the evolution of the band structure of C‐GeTe upon medium‐entropy alloying. Based on the similar variation trend, we intend to particularly focus on the spin‐up polarized band structure, which operates at working temperatures. It is observed that Mn doping doubles the *E*_g_. With Ge sites substituted by Cd, the *E*_g_ of C‐GeTe is further slightly increased. It is noteworthy that Cd doping gives rise to an impurity band close to the conduction band edge (cf. the blue band in Figure 3f), which could serve as deep levels and regulate the minority carriers.^[^ ^53^ ^]^ Despite sole Mn or Cd doping GeTe alloys were previously reported,^[^ ^27^, ^43^, ^44^, ^45^ ^]^ only the synergy of enlarged *E*_g_ and deep levels by (Mn, Pb, Sb, Cd) co‐alloying could effectively suppress the bipolar effect at high temperatures, which will be discussed later.

**Figure 4:**
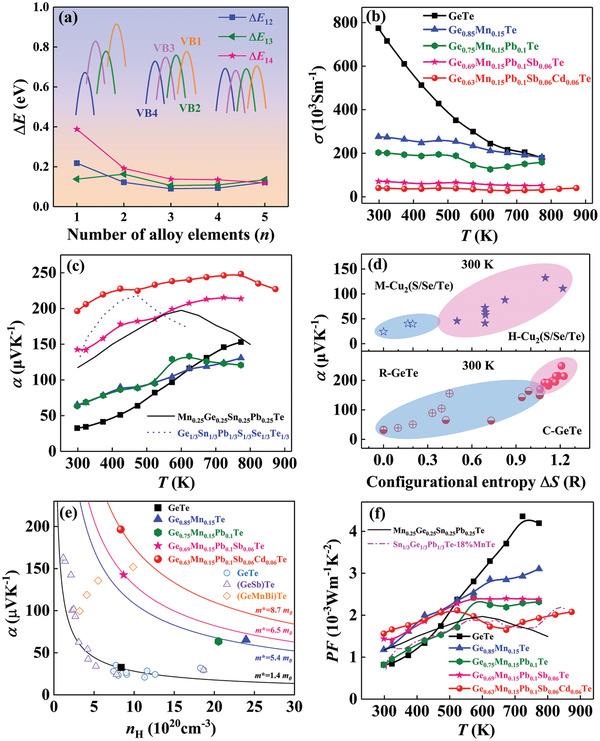
a) The energy offset Δ*E* of four valence bands for GeTe‐based alloys. Temperature dependence of b) electrical conductivity, c) Seebeck coeffcient, and f) power factor for GeTe‐based alloys.^[^ ^11^, ^54^ ^]^ d) Room‐temperature *α* as a function of Δ*S* for GeTe‐based alloys in comparison with literature data.^[^ ^11^, ^27^ ^]^ The dashed lines are guides to the eyes. e) Room‐temperature *α* as a function of *n*_H_ for GeTe‐based alloys in comparison with literature data.^[^ ^25^, ^44^ ^]^

Medium‐entropy alloying is beneficial to attain decent *μ*_H_, however, Mn alloying significantly flattens the VBs and hence diminishes the *μ*_H_. Zheng et. al. found that the *μ*_H_ was drastically reduced from 54.2 cm^2^ V^–1^ s^–1^ for binary GeTe to 4.4 cm^2^ V^–1^ s^–1^ for ternary Ge_0.85_Mn_0.15_Te as the density‐of‐state effective mass m* increased from 1.4 *m*_0_ to 6.2 *m*_0_.^[^ ^43^ ^]^ The subsequent Pb, Sb, and Cd alloying roughly maintain the VBs flattening. More importantly, (Mn, Pb, Sb, Cd) co‐alloying substantially reduces the Δ*E* with respect to four VBs. As shown in **Figure** **4**a, the Δ*E* between VB1 (G‐A direction) and VB2 (X‐G direction) drops from 0.22 eV for GeTe to 0.09 eV for Ge_0.75_Mn_0.15_Pb_0.1_Te, and then slightly increases to 0.12 eV for Ge_0.63_Mn_0.15_Pb_0.1_Sb_0.06_Cd_0.06_Te. A similar trend is observed for the Δ*E*_13_ between VB1 and VB3 (M‐X direction). More importantly, the Δ*E*_14_ between VB1 and VB4 (G‐M direction) monotonously decreases from 0.39 eV for GeTe to 0.12 eV for Ge_0.63_Mn_0.15_Pb_0.1_Sb_0.06_Cd_0.06_Te. To our best knowledge, it is the first time that the four VBs convergence is reported in GeTe‐based alloys, which is not be found in sole Mn/Pb/Sb/Cd doped GeTe.^[^ ^24^, ^25^, ^27^, ^43^, ^44^ ^]^ Such multivalence bands convergence, combined with the band flattening, can substantially enhance *α*.^[^ ^10^, ^27^, ^43^ ^]^

### 2.4 State‐Of‐The‐Art *zT* Values by Entropy Engineering

Based on the analysis of depressed phase transition, formed multiscale microstructures, and band structure evolution, an immediate question arises as to whether our GeTe‐based MEAs exhibit more superior TE performance than corresponding LEAs and HEAs. As plotted in Figure 4b, medium‐entropy alloying leads to a reduction in *σ* by the order of magnitude that is mainly attributed to the seriously degraded *μ*_H_ stemming from the band flattening and the enhanced alloy scattering. The degraded *σ* needs to be counteracted by appreciably improved *α* through band engineering.^[^ ^14^ ^]^

Figure 4c clearly indicates the *α*‐enhancement over the entire temperature range with increasing Δ*S*, particularly on the low‐temperature side. Typically, Ge_0.63_Mn_0.15_Pb_0.1_Sb_0.06_Cd_0.06_Te has *α* = 197 µV K^–1^ at 300 K, which rapidly increases to 248 µV K^–1^ at 773 K and mildly decreases to 227 µV K^–1^ at 873 K. Such *α* is not only much larger than that of binary GeTe, but also superior to those GeTe‐based HEAs.^[^ ^11^, ^40^, ^54^ ^]^

In view of the similar *n*_H_ between GeTe and Ge_0.63_Mn_0.15_Pb_0.1_Sb_0.06_Cd_0.06_Te, the unusual *α*‐enhancement in GeTe‐based alloys is mainly attributed to the following two reasons: i) medium‐entropy alloying enhances the crystal structure symmetry and hence the *N*_V_, contributing to the substantially increased *α*, especially in the low temperature range (Figure 4d).^[^ ^43^ ^]^ Similar phenomena can be observed in Cu_2_(Te/Se/S)^[^ ^11^ ^]^ and Cu_7_P(Se/Te)_6_.^[^ ^7^ ^]^ ii) (Mn, Pb, Sb, Cd) co‐alloying promotes multivalence band convergence and band flattening, resulting in remarkable enhancement in m* and thereupon *α*.

The well‐established Pisarenko relation between *α* and *n*_H_ can offer insight into the band structure variation based on the single parabolic band model (SPB) (Figure 4e).^[^ ^43^ ^]^ The Mn, (Mn, Pb), (Mn, Pb, Sb), and (Mn, Pb, Sb, Cd) alloyed samples present much larger m* than pristine GeTe. Specifically, the m* sharply increases from 1.4 *m*_0_ for binary GeTe to 5.4 *m*_0_ for Ge_0.75_Mn_0.15_Pb_0.1_Te, and further to 8.7 *m*_0_ for Ge_0.63_Mn_0.15_Pb_0.1_Sb_0.06_Cd_0.06_Te. Therefore, the substantially enlarged m* owing to the phase and band engineering leads to the remarkably increased *α* in the entire temperature range.

In addition, the bipolar effect has an important influence on the high temperature TE performance.^[^ ^53^ ^]^ It can be found that the downturn in the plot of *α* versus *T* (400–600 K) at high temperatures for GeTe HEAs including Ge_0.25_Sn_0.25_Pb_0.25_Mn_0.25_Te^[^ ^11^ ^]^ and Ge_1/3_Sn_1/3_Pb_1/3_Te_1/3_Se_1/3_S_1/3_,^[^ ^40^ ^]^ which is an indicator of the detrimental bipolar effect (Figure 4c). In contrast, no obvious contribution from bipolar effect is traced until 773 K for our Ge_0.63_Mn_0.15_Pb_0.1_Sb_0.06_Cd_0.06_Te sample as signified by the temperature‐dependent *α*. This marked difference is ascribed to the larger *E*_g_ and induced deep levels in our (Mn, Pb, Sb, Cd) co‐alloyed sample, effectively mitigating the intrinsic conduction.

The balance between the enhanced *α* and the declined *μ*_H_ determines the variation of PF with increasing Δ*S* (Figure 4f). Interestingly, the room‐temperature PF substantially ascends from 0.82 × 10^–3^ W m^–1^ K^–1^ for the pristine GeTe to 1.56 × 10^–3^ W m^–1^ K^–1^ for the Ge_0.63_Mn_0.15_Pb_0.1_Sb_0.06_Cd_0.06_Te, which is favorable for attaining good average *zT*_ave_. Although the high‐temperature PF falls with increasing number of solid solution components due to the decreased *μ*_H_, the PF at 873 K is still 2.1 × 10^–3^ W m^–1^ K^–1^ for the Ge_0.63_Mn_0.15_Pb_0.1_Sb_0.06_Cd_0.06_Te sample. The PF in this work is much higher than those of GeTe‐based HEAs including Ge_0.25_Sn_0.25_Pb_0.25_Mn_0.25_Te,^[^ ^11^ ^]^ Ge_1/3_Sn_1/3_Pb_1/3_Te_1/3_Se_1/3_S_1/3_,^[^ ^40^ ^]^ and Sn_1/3_Ge_1/3_Pb_1/3_Te‐18%MnTe.^[^ ^54^ ^]^

**Figure 5:**
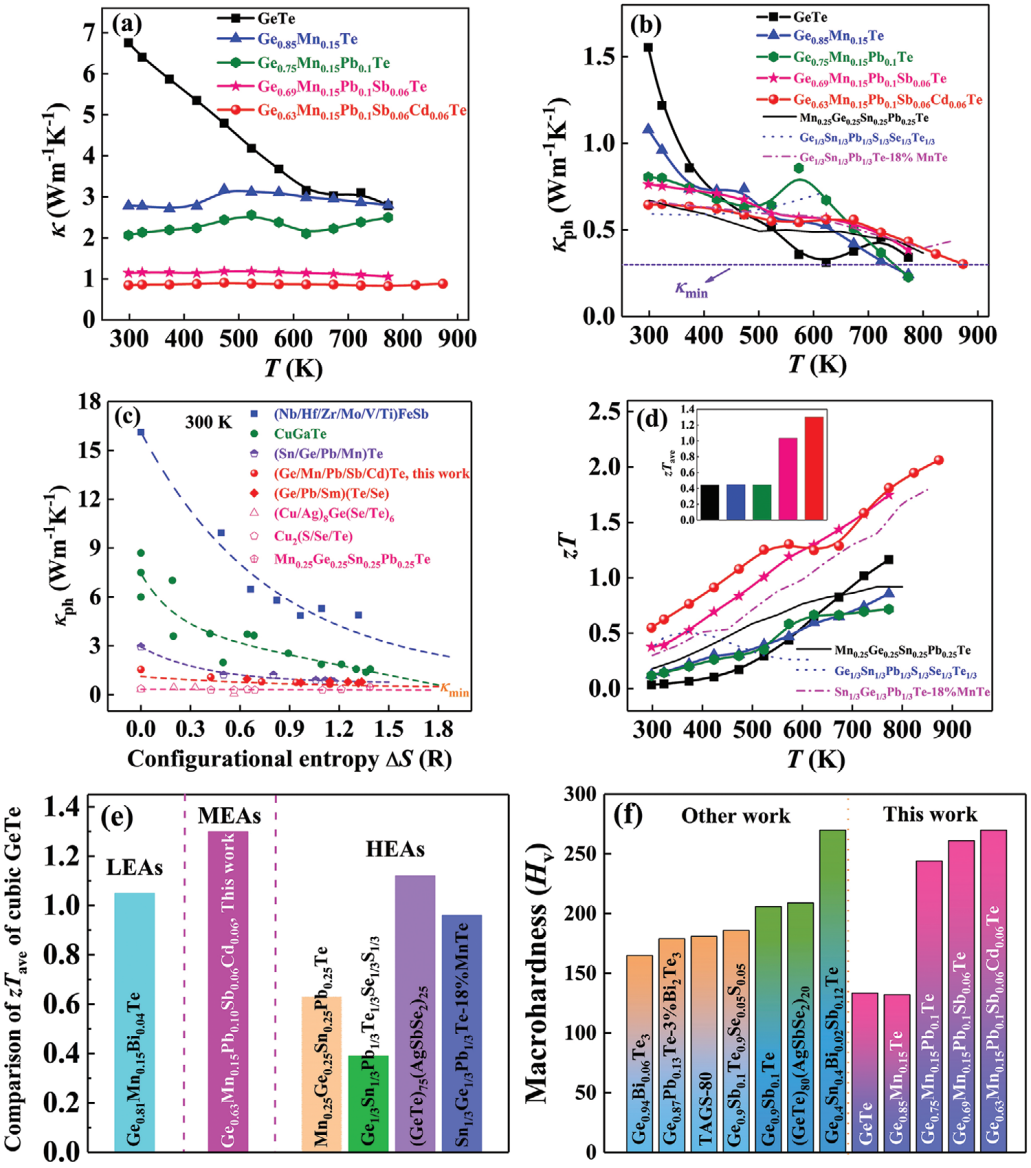
Temperature dependences of a) total thermal conductivity, b) lattice thermal conductivity, and d) *zT* for GeTe‐based alloys. The inset in (d) is the average *zT*_ave_ values in the measured temperature range.^[^ ^11^, ^40^, ^54^ ^]^ c) Room‐temperature *κ*_ph_ as a function of Δ*S* for GeTe‐based alloys in comparison with literature data.^[^ ^10^, ^11^, ^55^, ^56^, ^57^ ^]^ The dashed lines are guides to the eyes. e) Comparison of average *zT*_ave_ values of cubic GeTe with different compositions.^[^ ^11^, ^40^, ^41^, ^44^, ^54^ ^]^ f) Room‐temperature Vickers hardness *H*_v_ of GeTe‐based alloys in comparison with literature data.^[^ ^26^, ^41^, ^58^, ^59^, ^60^ ^]^

For the high *κ* of binary GeTe, increasing Δ*S* is an obvious choice to concurrently decrease the *κ*_el_ (owing to the decreased *σ*) and *κ*_ph_ (**Figure** **5**a,b and Figure S21, Supporting Information). Similar to the variation of *α* versus *T* (Figure 4c), our Ge_0.63_Mn_0.15_Pb_0.1_Sb_0.06_Cd_0.06_Te sample has large *E*_g_ and deep levels and thereupon suppressed bipolar effect at high temperature, leading to sustained fall in *κ*_ph_ with increasing *T* (Figure 5b). Furthermore, it is more concerned whether our GeTe‐based MEAs could obtain the similar low *κ*_ph_ compared to HEAs. As mentioned above, medium‐entropy alloying‐induced multiscale microstructures, including atomic‐scale substitutions, interstitials, dislocation arrays, ordered Ge vacancies, sub‐nanoscale strain clusters, and micron‐scale Mn precipitations, result in the largely depressed *κ*_ph_. The gradual saturation of phonon scattering has been observed as the number of components increases from one to five (Figure 5c), limiting the persistent reduction in *κ*_ph_. Surprisingly, the minimum *κ*_ph_ for the (Mn, Pb, Sb, Cd) co‐alloyed sample is only 0.30 W m^−1^ K^−1^ at 873 K; this is much lower than the amorphous limit of GeTe (0.4 W m^−1^ K^−1^).^[^ ^33^ ^]^ Note that the derived *κ*_ph_ in this work is nearly the same as those of GeTe‐based HEAs, such as Ge_0.25_Sn_0.25_Pb_0.25_Mn_0.25_Te,^[^ ^11^ ^]^ Ge_1/3_Sn_1/3_Pb_1/3_Te_1/3_Se_1/3_S_1/3_,^[^ ^40^ ^]^ and Sn_1/3_Ge_1/3_Pb_1/3_Te‐18%MnTe.^[^ ^54^ ^]^ It is worth re‐emphasizing that medium‐entropy alloying is sufficient to suppress the *κ*_ph_ to the glass limit of a solid for GeTe,^[^ ^55^ ^]^ SnTe,^[^ ^10^ ^]^ and CuGaTe_2_^[^ ^56^ ^]^ with moderate initial *κ*_ph_. Unlike half‐Heusler alloys with high initial *κ*_ph_, only high‐entropy alloying could reduce the *κ*_ph_ to their amorphous limit (Figure 5c).^[^ ^57^ ^]^ With respect to Cu_2_(S/Se/Te) and (Cu/Ag)_8_Ge(Se/Te)_6_ with intrinsic ultralow *κ*_ph_,^[^ ^11^ ^]^ there has weak impact of Δ*S* on *κ*_ph_. Indeed, in supplemental studies we have tried six alloy elements, including Ge_0.64‐_ *_m_*Mn_0.15_Pb_0.1_Sb_0.06_Cd_0.05_In*_m_*Te, and Ge_0.64‐_ *_n_*Mn_0.15_Pb_0.1_Sb_0.06_Cd_0.05_Zn*_n_*Te series (Figure S23, Supporting Information), which cannot induce the further reduction in *κ*_ph_. Despite the *α* has been enhanced upon further In or Zn alloying, the continuous degradation of *μ*_H_ and hence PF excludes any TE promise by alloying more elements.

Through the medium‐entropy alloying‐driven synergy of phase, phonon, and band engineering, *zT* is tremendously enhanced over the entire temperature range (Figure 5d). As Δ*S* increases, the highest *zT* reaches 2.1 at 873 K for Ge_0.63_Mn_0.15_Pb_0.1_Sb_0.06_Cd_0.06_Te, almost 178% higher than that of binary GeTe. Furthermore, the maximum *zT* in this study is much higher than those of GeTe‐based HEAs.^[^ ^11^, ^40^, ^54^ ^]^ Surprisingly, the room‐temperature *zT* is substantially improved from 0.04 for pristine GeTe to 0.55 for Ge_0.63_Mn_0.15_Pb_0.1_Sb_0.06_Cd_0.06_Te; this is essential to attaining high average *zT*_ave_. Consequently, the Ge_0.63_Mn_0.15_Pb_0.1_Sb_0.06_Cd_0.06_Te sample yields an average *zT*_ave_ of 1.3 in the range of 300–873 K, which is the highest value reported for pure cubic GeTe‐based alloys (Figure 5e).^[^ ^11^, ^40^, ^41^, ^44^, ^54^, ^58^ ^]^ As a result, the concurrently depressed phase transition and high *zT* > 2 is for the first time reported in GeTe‐based alloys, which may pave the way for their practical applications. Furthermore, we tune the *n*_H_ by Ge self‐doping in the subsequent Ge_0.63 +_ *_t_*Mn_0.15_Pb_0.1_Sb_0.06_Cd_0.06_Te series (Figure S24, Supporting Information). However, the further improvement of *zT* is failed regardless of Ge excess or short, indicating that the fully optimized *n*_H_ is indeed achievable in the present work.

It is known that MEAs possess better mechanical properties in comparison with LEAs that make them attractive for structural applications.^[^ ^15^ ^]^ The Vickers hardness *H*_V_ ≈ 134 for undoped GeTe is less satisfactory owing to the presence of Ge vacancies (Figure 5f). Upon medium‐entropy alloying, the *H*_V_ is significantly enhanced to 270 for Ge_0.63_Mn_0.15_Pb_0.1_Sb_0.06_Cd_0.06_Te; this is a record high value among all the previously reported GeTe‐based alloys.^[^ ^26^, ^41^, ^59^, ^60^ ^]^ Although a similar high *H*_V_ is reported for Ge_0.4_Sn_0.4_Bi_0.02_Sb_0.12_Te, our sample possesses higher *zT* and average *zT*_ave_.^[^ ^58^ ^]^ The efficacy of medium‐entropy thermoelectrics by integrating phase, phonon, and band engineering is thereby confirmed.

## 3 Conclusion

In summary, we go beyond the traditional low‐entropy thermoelectrics and the emerging high‐entropy thermoelectrics to exploit the power of medium‐entropy thermoelectrics in order to substantially minimize the lattice thermal conductivity and retain good electrical properties. GeTe‐based alloys with moderate lattice thermal conductivity and crystal symmetry have been selected as paradigm to verify the efficacy of this novel strategy. Owing to the sufficiently high configuration entropy, the rhombohedral to cubic phase transition, which limits their practical applications, was completely suppressed above 300 K. A lower‐than‐amorphous‐limit lattice thermal conductivity of 0.30 W m^−1^ K^−1^ at 873 K was attained in cubic GeTe‐based alloys. Meanwhile, (Mn, Pb, Sb, Cd) co‐doping facilitated the band convergence and increased the band effective mass, all of which substantially improved the Seebeck coefficient and compensated for the loss of carrier mobility. For the first time, a state‐of‐the‐art *zT* of 2.1 at 873 K, high average *zT*_ave_ value of 1.3 between 300 and 873 K and record high Vickers hardness of 270 were simultaneously attained in cubic Ge_0.63_Mn_0.15_Pb_0.1_Sb_0.06_Cd_0.06_Te with no phase transition. These results amount to a breakthrough in the burgeoning field of entropy engineering by introducing the paradigm‐shifting “medium‐entropy thermoelectrics.”

## Conflict of Interest

The authors declare no conflict of interest.

## Supporting information

Supporting Information

## Data Availability

Data available on request from the authors. The data that support the findings of this study are available from the corresponding author upon reasonable request.
