# Supplementary material for: Medium Entropy‐Enabled High Performance Cubic GeTe Thermoelectrics
Source: Adv Sci (Weinh). 2021 May 6;8(12):2100220. doi: 10.1002/advs.202100220 (PMC8224415; doi:10.1002/advs.202100220)
Supplement: Supplementary file 1 — Supporting Information [file ADVS-8-2100220-s001.pdf]

WILEY-VCH

© 2021 Wiley-VCH GmbH

ADVANCED SCIENCE
Open Access

Supporting Information

for *Adv. Sci.,* DOI: 10.1002/advs.202100220

Medium Entropy-enabled High Performance Cubic GeTe Thermoelectrics

Shizhen Zhi, Jibiao Li, Lipeng Hu, Junqin Li, Ning Li, Haijun Wu, Fusheng Liu, Chaohua Zhang, Weiqin Ao, Heping Xie, Xinbing Zhao, Stephen John Pennycook, and Tiejun Zhu**

Supporting Information

Medium Entropy-enabled High Performance Cubic GeTe Thermoelectrics

Shizhen Zhi,[a#] Jibiao Li,[b,c#] Lipeng Hu,[a] Junqin Li,[a] Ning Li,[e] Haijun Wu,[d,e*] Fusheng Liu,[a] Chaohua Zhang,[a] Weiqin Ao,[a] Heping Xie,[a] Xinbing Zhao,[f] Stephen John Pennycook,[e] and Tiejun Zhu[f*]*

S. Z. Zhi, Dr. L. P. Hu, Prof. J. Q. Li, Prof. F. S. Liu, Prof. C. H. Zhang, Prof. W. Q. Ao, Prof. H. P. Xie
College of Materials Science and Engineering, Shenzhen Key Laboratory of Special Functional Materials, Guangdong Research Center for Interfacial Engineering of Functional Materials, Guangdong Provincial Key Laboratory of Deep Earth Sciences and Geothermal Energy Exploitation and Utilization, Institute of Deep Earth Sciences and Green Energy, Shenzhen University, Shenzhen 518060, China
E-mail: hulipeng@szu.edu.cn

Dr. J. B. Li
Center for Materials and Energy (CME) and Chongqing Key Laboratory of Extraordinary Bond Engineering and Advanced Materials Technology (EBEAM), Yangtze Normal University, Chongqing 408100, China

Dr. J. B. Li
Institute for Clean Energy and Advanced Materials, Southwest University, Chongqing 400715, China

Prof. H. J. Wu
State Key Laboratory for Mechanical Behavior of Materials, Xi'an Jiaotong University, Xi'an 710049, China
E-mail: wu.haijun@u.nus.edu

N. Li, Prof. H. J. Wu, Prof. S. J. Pennycook
Department of Materials Science and Engineering, National University of Singapore, Singapore 117575, Singapore

Prof. X. B. Zhao, Prof. T. J. Zhu
State Key Laboratory of Silicon Materials and School of Materials Science and Engineering, Zhejiang University, Hangzhou 310027, China
E-mail: zhutj@zju.edu.cn

Experimental Section

1. Sample Synthesis and Preparation. High-purity elemental chunks of Ge, Mn, Pb, Sb, Bi, Cd, Zn, In, and Te, purchased from Beijing Yipin Chuancheng Technology Co., Ltd. (YPCC), were used as raw materials. Appropriate quantities of elements were weighed and mixed according to the nominal compositions of GeTe, $Ge_{0.85}Mn_{0.15}Te$, $Ge_{0.75}Mn_{0.15}Pb_{0.1}Te$, $Ge_{0.75-x}Mn_{0.15}Pb_{0.1}Sb_xTe$ (x = 0.06, 0.08, 0.10, 0.12), $Ge_{0.75-y}Mn_{0.15}Pb_{0.1}Bi_yTe$ (y = 0.04, 0.05, 0.06, 0.07, 0.08, 0.10, 0.12), $Ge_{0.69-z}Mn_{0.15}Pb_{0.1}Sb_{0.06}Cd_zTe$ (z = 0.04, 0.05, 0.06, 0.08, 0.10), $Ge_{0.64-m}Mn_{0.15}Pb_{0.1}Sb_{0.06}Cd_{0.05}In_mTe$ (m = 0.005, 0.01, 0.02), $Ge_{0.64-n}Mn_{0.15}Pb_{0.1}Sb_{0.06}Cd_{0.05}Zn_nTe$ (n = 0.03, 0.05, 0.07), $Ge_{0.63+t}Mn_{0.15}Pb_{0.1}Sb_{0.06}Cd_{0.06}Te$ (t = -0.01, -0.02, -0.03, 0.01, 0.02, 0.03), and then sealed into a 20 mm-diameter quartz tube at 10^{-3} Pa. These tubes were slowly heated up to 1323 K, dwelt for 10 h, and quenched at 873 K by water. These ingots were ball milled (MSK-SFM-3, MTI Corporation) at 1200 rpm for 20 min to fine powders in vacuum. The powders were spark plasma sintering (SPS) into a cylinder in a 20 mm graphite die at 873 K for 5 min under 50 MPa to obtain the high-density bulk samples.

2. Sample Characterization. The powder X-ray diffraction data were obtained on a SmartLab diffractometer with the Cu Kα radiation operating at 40 KV × 200 mA. The lattice parameters were refined by the Rietveld method via GSAS software with EXPGUI interface.[1,2] The microstructures were inspected by Scanning electron microscopy (SEM, Hitachi SU-70, Japan) and the chemical compositions were analyzed by energy dispersive spectrometer (EDS). (Scanning) transmission electron microscopy (STEM and TEM) studies were conducted using a JEOL ARM200F atomic resolution analytical electron microscope installed in the National University of Singapore equipped with a cold field-emission gun, a new ASCOR 5th order aberration corrector and Gatan OneView camera. The specimens were

prepared by conventional standard methods, that is, cutting, grinding, dimpling, polishing and Ar-ion milling with a liquid nitrogen cooling stage (Fischione M1051 TEM Mill).

The phase transition temperature T_0 of the samples was measured by using a differential scanning calorimeter (DSC TAQ2000, USA). The longitudinal v_l and transverse v_t components of the sound velocity were measured using an ultrasonic pulse receiver (MS-100, Ritec Lab, France). The mean phonon sound v_m, Poisson's ratio v_P, Gruneisen parameters γ, and the phonon mean free path l_{ph} were evaluated from the measured sound velocity by using the following formulas:[3]

$$v_m = \left\{ \frac{1}{3} \left(\frac{1}{v_l^3} + \frac{2}{v_t^3} \right) \right\}^{(-1/3)} \tag{1}$$

$$v_p = \frac{1-2(v_t/v_l)^2}{2-2(v_t/v_l)^2} \tag{2}$$

$$\gamma = \frac{3}{2}\left(\frac{1+v_p}{2-3v_p} \right) \tag{3}$$

$$\kappa_{ph} = \frac{1}{3} C_V v_m l_{ph} \tag{4}$$

where C_V is the specific heat. To study the mechanical properties, the Vickers Hardness H_v was carried out at 300 K on the HV-30Z.

3. Transport Property Measurements. Thermal conductivity was calculated through $\kappa = D\rho C_P$.The thermal diffusivity D was measured with a Netzsch LFA 467 HT laser flash apparatus and the density ρ was estimated by an Archimedes method. Considering the large challenge of accurate measurement of the specific heat C_P, here the C_P was determined using the Dulong-Petit law, which is usually used in previously reported GeTe-based alloys.[4-12] In addition, most of the literature reporting a high zT in GeTe uses a C_P at or very close to the

Dulong-Petit limit.[4,7,13-16] The electrical conductivity σ and the Seebeek coefficient S were simultaneously measured under helium atmosphere by using ZEM-2 (Ulvac-Riko®, Japan). The room temperature Hall coefficient R_H was measured on a PPMS system (Quantum Design®) with magnetic fields sweeping from -5T to 5T. The carrier concentration n_H and Hall mobility μ_H were estimated by $n_H = 1/eR_H$ and $\mu_H = \sigma R_H$, respectively.

4. Electronic Band Structure Calculations. Quantum mechanical calculations were performed by using the QUANTUM ESPRESSO package which is based on spin polarized density functional theory (DFT). Electron-ion interactions were modeled by the use of the projector augmented wave (PAW) method. Electron exchange and correlation effects were represented by the Perdew-Burke-Ernzerhof form (PBE) with the full relativistic method. The spin-orbital couplings (SOC) are taken into consideration throughout all the calculations. Kohn-Sham orbitals were expanded in a plane wave basis set with a cutoff energy of 49 Ry and 599 Ry for the charge density cutoff. The Brillouin zone integration was performed by the Gaussian special point technique, with a smearing parameter of 0.02 Ry and k-point meshes of 4×4×1. In addition to the general band structures, band structures are also projected onto orbital contributions of selected atoms of our interests. Spin density and Kohn-Sham orbitals at the conduction band edge (CBE) and valence band edge (VBE) at specific K points are obtained in successive planes cutting through the atomic layers in the unit cell.

The rhombohedral GeTe (R-GeTe) was modelled by a hexagonal unit cell containing 48 atoms ($Ge_{24}Te_{24}$). The cubic GeTe (C-GeTe) was represented by a hexagonal unit cell which contains 54 atoms in alternative atomic layers as described by the chemical formulas of $Ge_{27}Te_{27}$. The periodic boundary conditions were applied to the model structure in geometry optimization. The doped C-GeTe materials were modeled by replacing one Ge atom by one dopant atom (e.g. Mn, Pb, Sb, Cd). A series of doped materials are modeled by successive doping in the sequence of Mn, Pb, Sb, and Cd atoms. Therefore, hexagonal crystals including

$Ge_{24}Mn_3Te_{27}$, $Ge_{22}Mn_3Pb_2Te_{27}$, $Ge_{21}Mn_3Pb_2Sb_1Te_{27}$, $Ge_{20}Mn_3Pb_2Sb_1Cd_1Te_{27}$ are used to simulate the successively doped materials in different stages. All sites of replacement are considered to obtain the global stable structures in a large amount of relaxation calculations. Structure optimizations were performed with all the atoms were fully relaxed until the Hellmann-Feynman forces were lower than 0.001 Ry/a.u. A calculation was considered converged when the energy change per atom was less than 10^{-5} Ry and the mean displacement less than 0.001 Å. The convergence threshold for SCF calculations was set to 10^{-5} Ry. The force convergence threshold for geometry optimizations was set to 0.02 eVÅ^{-1}.

Table S1. The solubility limit of Mn, Pb, Sb, Cd in binary GeTe.

**Elements**	**Mn**	**Pb**	**Sb**	**Cd**
Solubility	18%[17]	10%[18]	>15%[19]	10%[20]

Table S2 Longitudinal v_l, shear wave v_t and mean sound velocity v_m, phonon mean free path l_{ph}, and Gruneisen parameters γ of our GeTe-based alloys.

**Samples**	**v_l (ms^{-1})**	**v_t (ms^{-1})**	**v_m (ms^{-1})**	**l_{ph} (Å)**	γ
GeTe	3353	1913	2126	29.1	1.54
$Ge_{0.85}Mn_{0.15}Te$	3283	1713	1917	11.3	1.86
$Ge_{0.75}Mn_{0.15}Pb_{0.1}Te$	3200	1704	1904	8.7	1.79
$Ge_{0.69}Mn_{0.15}Pb_{0.1}Sb_{0.06}Te$	3300	18523	2062	7.5	1.60
$Ge_{0.63}Mn_{0.15}Pb_{0.1}Sb_{0.06}Cd_{0.06}Te$	3284	1848	2056	6.4	1.60

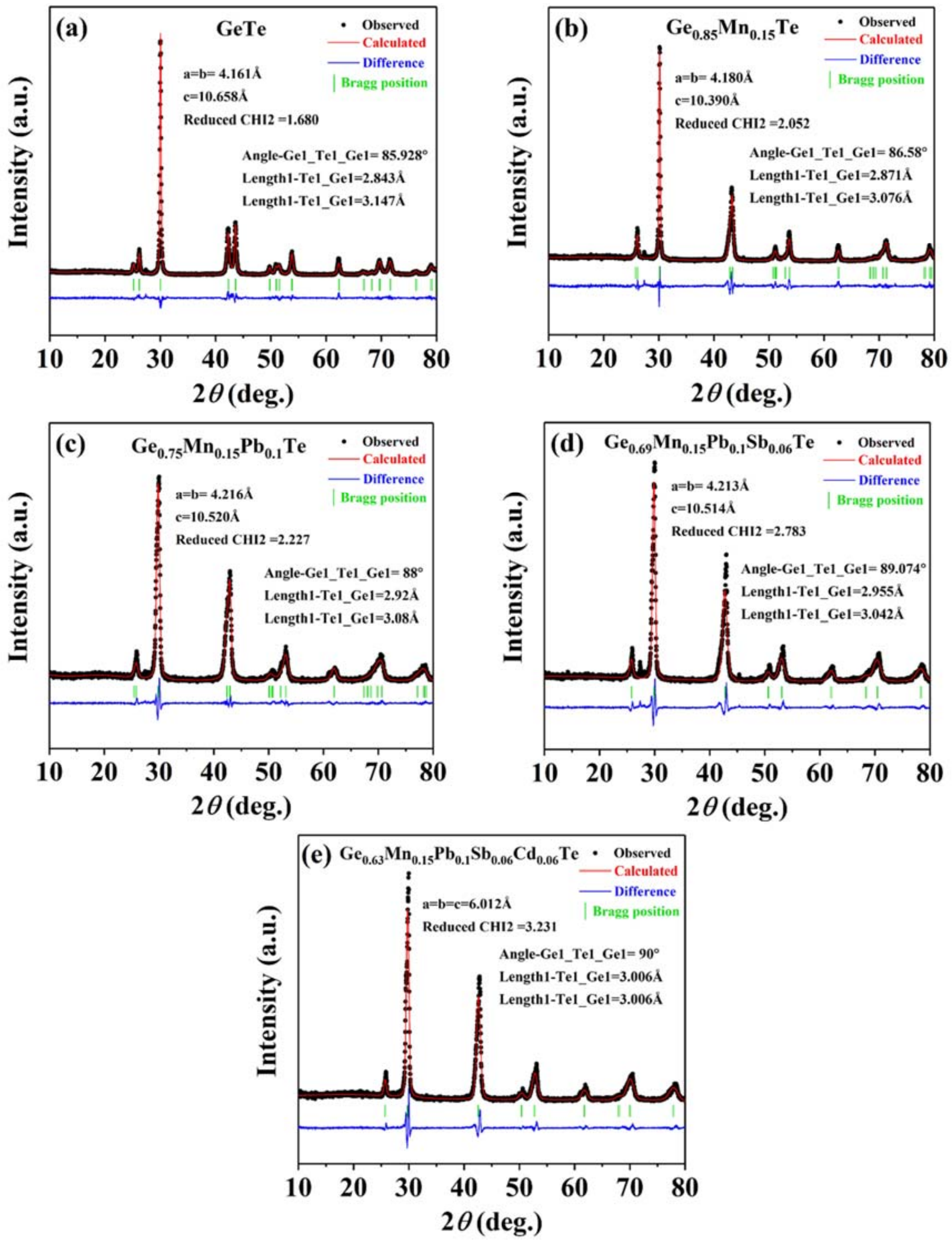

Figure S1. Rietveld refinement results of (a) GeTe, (b) $Ge_{0.85}Mn_{0.15}Te$, (c) $Ge_{0.75}Mn_{0.15}Pb_{0.1}Te$, (d) $Ge_{0.69}Mn_{0.15}Pb_{0.1}Sb_{0.06}Te$, and (e) $Ge_{0.63}Mn_{0.15}Pb_{0.1}Sb_{0.06}Cd_{0.06}Te$.

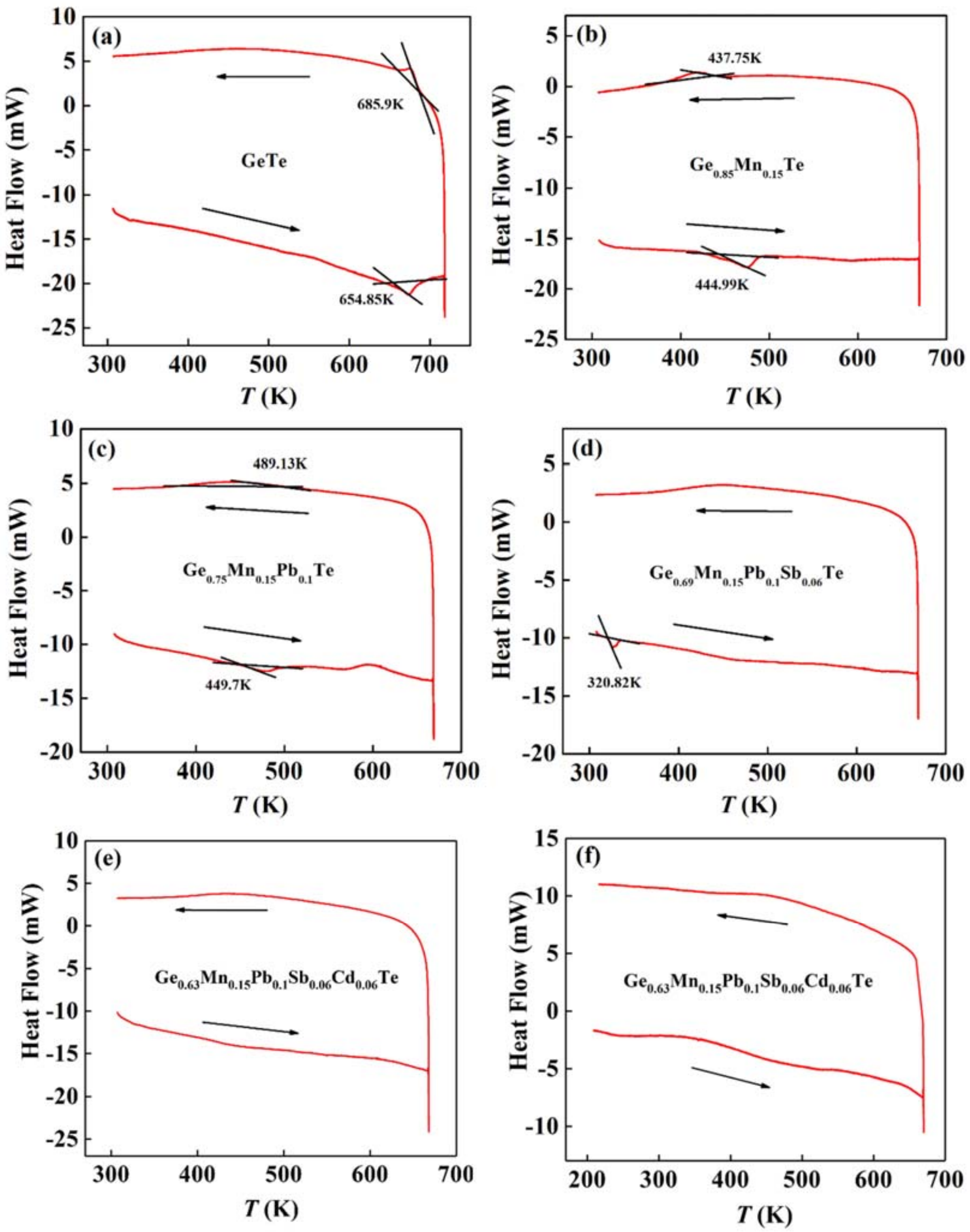

Figure S2. (a)-(e) DSC curves of GeTe, $Ge_{0.85}Mn_{0.15}Te$, $Ge_{0.75}Mn_{0.15}Pb_{0.1}Te$, $Ge_{0.69}Mn_{0.15}Pb_{0.1}Sb_{0.06}Te$, and $Ge_{0.63}Mn_{0.15}Pb_{0.1}Sb_{0.06}Cd_{0.06}Te$. (f) DSC curve between 190 K and 670 K of $Ge_{0.63}Mn_{0.15}Pb_{0.1}Sb_{0.06}Cd_{0.06}Te$.

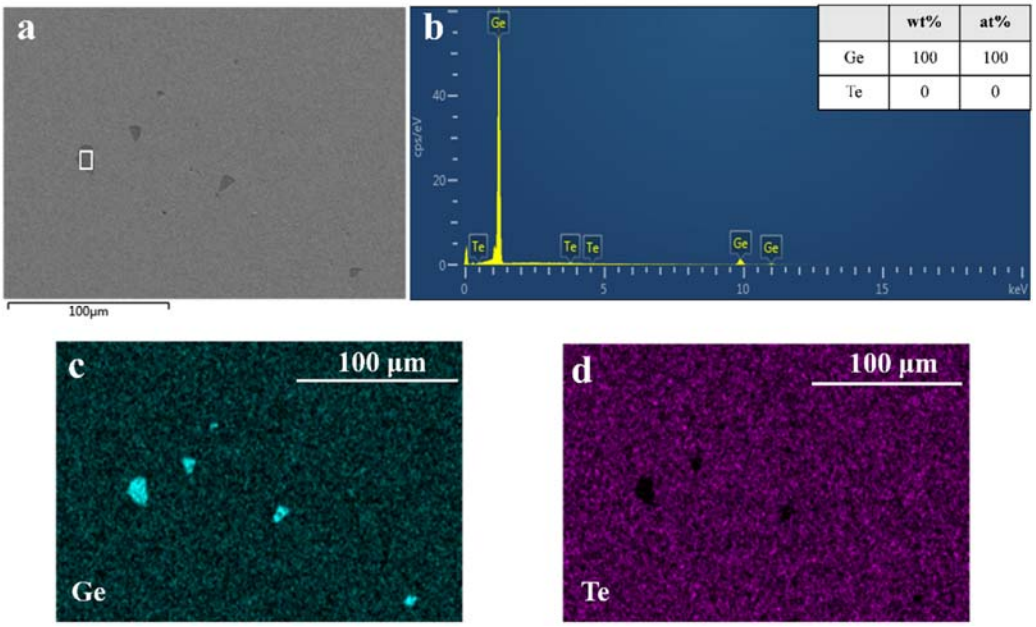

Figure S3. SEM and EDS images of GeTe sample, showing the Ge second phase.

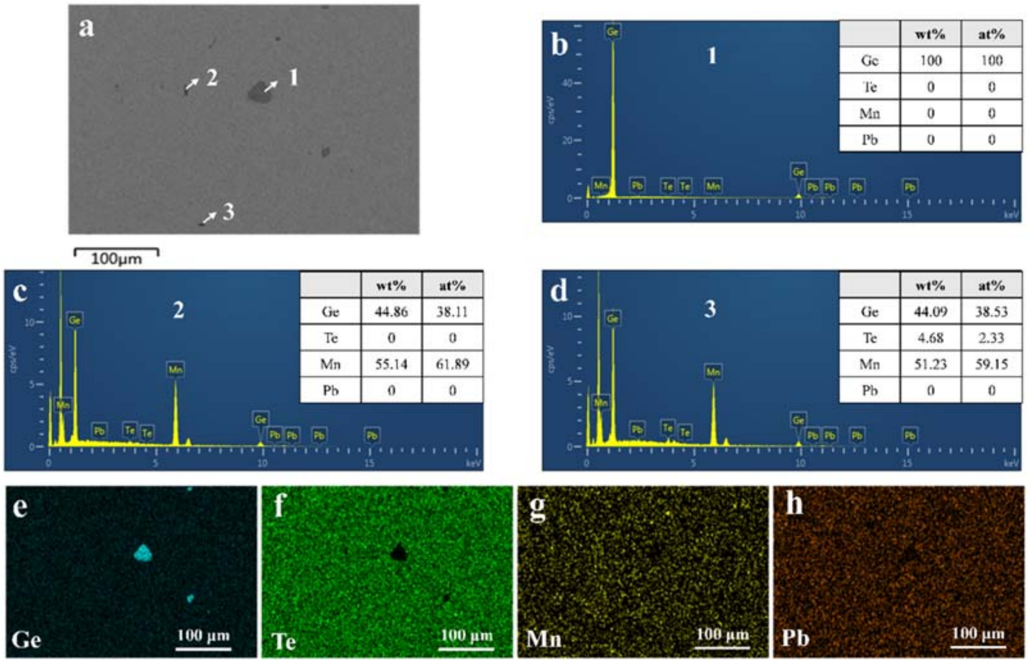

Figure S4. SEM and EDS images of $Ge_{0.75}Mn_{0.15}Pb_{0.1}Te$ sample, showing the Ge and Mn second phase.

Tiny amounts of Mn second phase was observed in the $Ge_{0.75}Mn_{0.15}Pb_{0.1}Te$ sample, which was not detected in the XRD pattern (Figure 1).

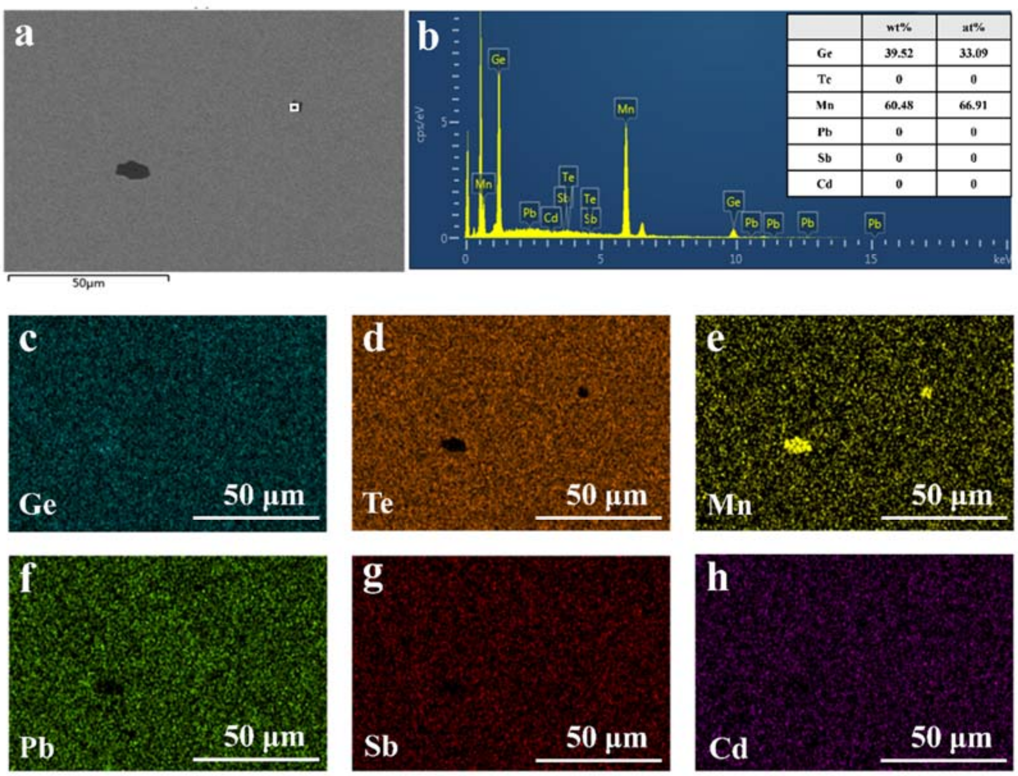

Figure S5. SEM and EDS images of $Ge_{0.63}Mn_{0.15}Pb_{0.1}Sb_{0.06}Cd_{0.06}Te$ sample, showing the Mn second phase.

The high-entropy effect also tends to expand the solubility limits of the specific elements.[21] Notably, binary GeTe is a highly degenerated *p*-type semiconductor with room-temperature n_H as high as 10^{21} cm^{-3}, stemming from the existence of Ge vacancies and Ge precipitations.[22] It can be seen that two weak peaks of Ge second phases at 27-27.5° and 45-45.5° are observed in pristine GeTe (Figure 1b). Besides, the scanning electron microscopy (SEM) and energy dispersive X-ray spectroscopy (EDS) results of GeTe sample indicate that the dark grey regions of several microns is Ge second phases (Figure S3, Supporting Information). Interestingly, the peaks of Ge phase become smaller and eventually disappear with increasing ΔS (Figure 1b and Figure S3-5, Supporting Information). For the multicomponent system, the Gibbs free energy of mixing ΔG is determined by the following equation:[23]

$$\Delta G = \Delta H - T\Delta S \quad (5)$$

where ΔH is the enthalpy of mixing. It can be found that if the ΔH keeps unchanged, a higher ΔS gives rise to a lower ΔG. Thus, *high-entropy effect* may promote the gradual dissolution of Ge precipitates in our GeTe-based MEAs, which in turn reduces the excessive concentration of Ge vacancies and hence n_H. Similarly, the solubility limit of Mn is substantially increased from ~13–15 mol% in binary SnTe to ~20 mol% in $(Sn_{0.7}Ge_{0.2}Pb_{0.1})_{1-x}Mn_{1.1x}Te$.[21]

Besides, tiny amounts of Mn second phase was still observed in the $Ge_{0.63}Mn_{0.15}Pb_{0.1}Sb_{0.06}Cd_{0.06}Te$ sample, which is associated with the formation of Mn interstitials (Figure 2).

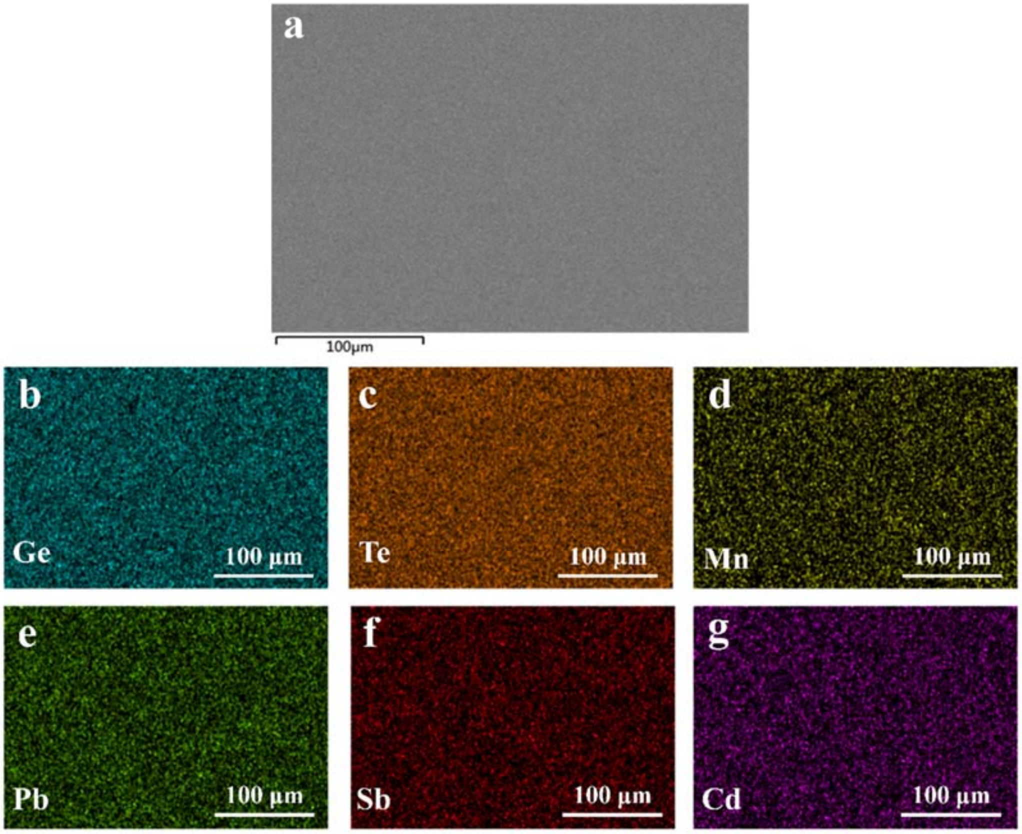

Figure S6. Elemental maps of $Ge_{0.63}Mn_{0.15}Pb_{0.1}Sb_{0.06}Cd_{0.06}Te$ obtained by EDS, indicating that all the elements are homogeneous in the matrix.

Electron probe microanalysis reveals that all elements are homogeneously distributed in the matrix for the $Ge_{0.63}Mn_{0.15}Pb_{0.1}Sb_{0.06}Cd_{0.06}Te$ sample.

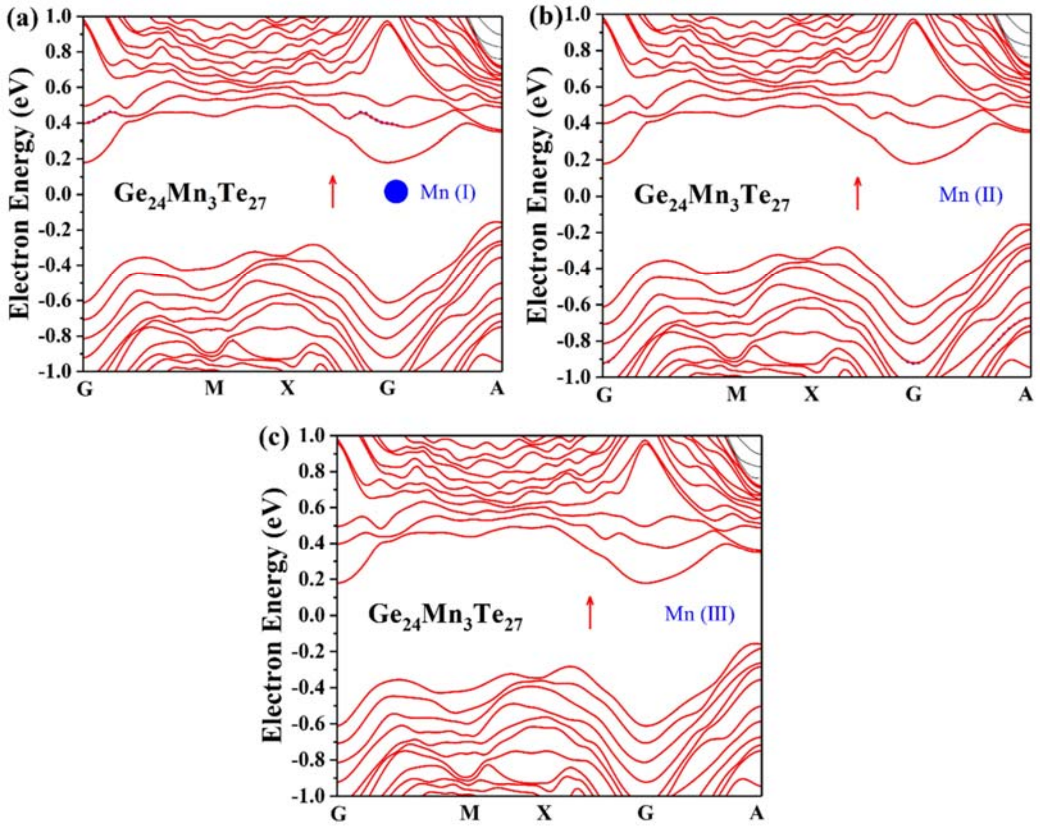

Figure S7. The electronic band structures (spin up) with projected elemental contributions of C-$Ge_{24}Mn_3Te_{27}$. The Fermi level is positioned at zero energy. The upward red arrow represents the spin up polarized electron band structures.

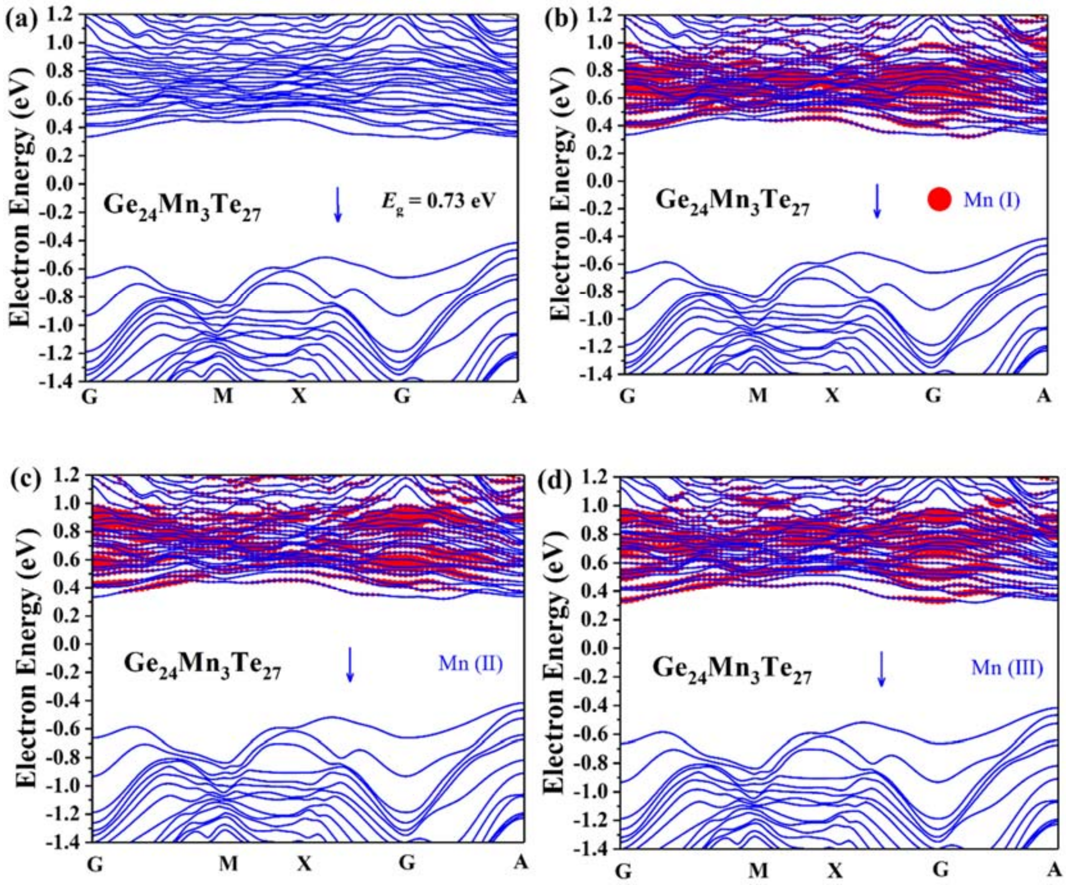

Figure S8. (a) Electron band structures (spin down) of C-$Ge_{24}Mn_3Te_{27}$. (b)-(d) The electronic band structures (spin down) with projected elemental contributions of C-$Ge_{24}Mn_3Te_{27}$. The Fermi level is positioned at zero energy. The downward blue arrow represents the spin up polarized electron band structures.

The Mn alloying exerts a significant impact on electronic structure controlling the TE performance. At the foremost, isolated Mn dimers in linear connection by the two ends are the favored local structural motifs in $Ge_{24}Mn_3Te_{27}$. The spin density reveals that two Mn atoms may exhibit super-ferromagnetic couplings in the majority spin (spin-up electrons), whereas the induced minority spin density (spin-down) is only present within the atomic Te planes. Secondly, the Mn doping creates two contrasting spin channels but only the spin up channel operates at working temperatures, given that spin flipping is forbidden in thermally activated processes. The spin up channel is of the semiconducting nature, whereas the spin down channel is govern by the insulating nature. Thus, $Ge_{24}Mn_3Te_{27}$ shows a strong preference of spin up electrons on electronic conducting via the bottom conduction band (BCB) and next

BCB band around the gamma, despite the fact that spin down electrons significantly contribute to the conduction band. Thirdly, the Mn doping in C-GeTe has significantly flattened the valence bands regardless of the spin up and spin down channels, thereby considerably slowed down the carrier mobility in the electronic transport by increasing the effective masses of holes. Moreover, the Mn doping in C-GeTe has doubled the band gap, suppressing the detrimental bipolar effect.

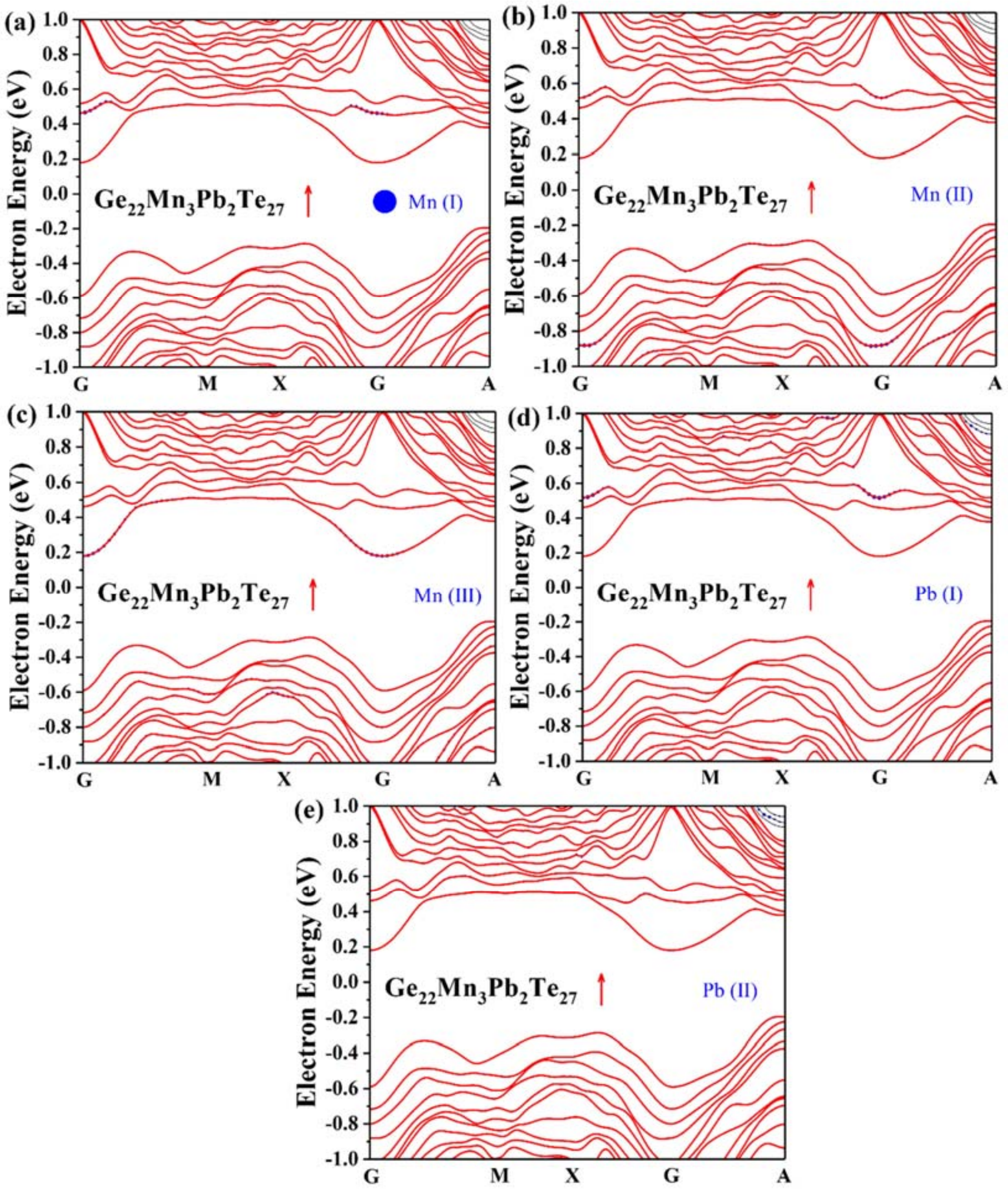

Figure S9. The electronic band structures (spin up) with projected elemental contributions of C-$Ge_{22}Mn_3Pb_2Te_{27}$. The Fermi level is positioned at zero energy. The upward red arrow represents the spin up polarized electron band structures.

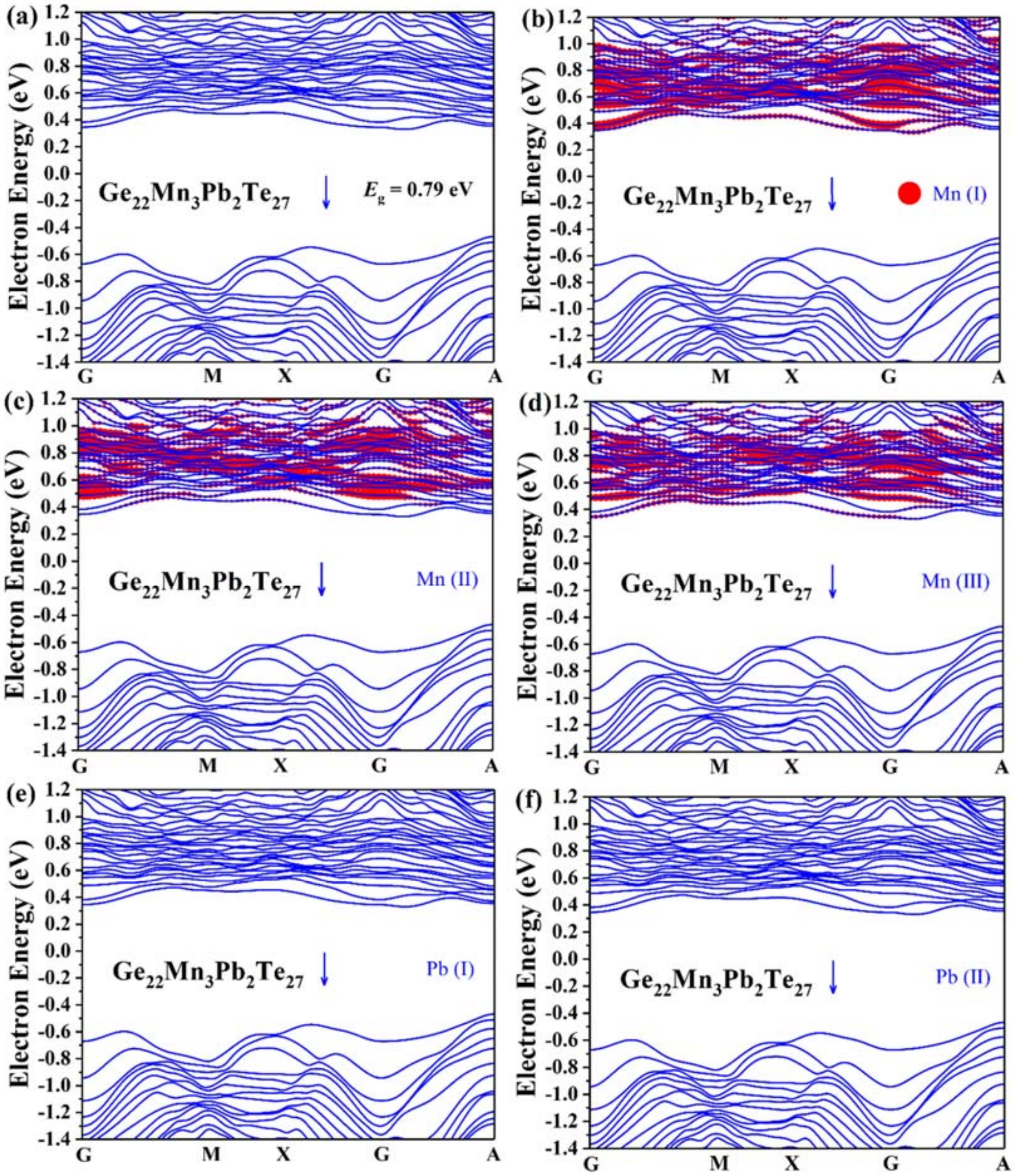

Figure S10. (a) Electron band structures (spin down) of C-$Ge_{22}Mn_3Pb_2Te_{27}$. (b)-(f) The electronic band structures (spin down) with projected elemental contributions of C-$Ge_{22}Mn_3Pb_2Te_{27}$. The Fermi level is positioned at zero energy. The downward blue arrow represents the spin up polarized electron band structures.

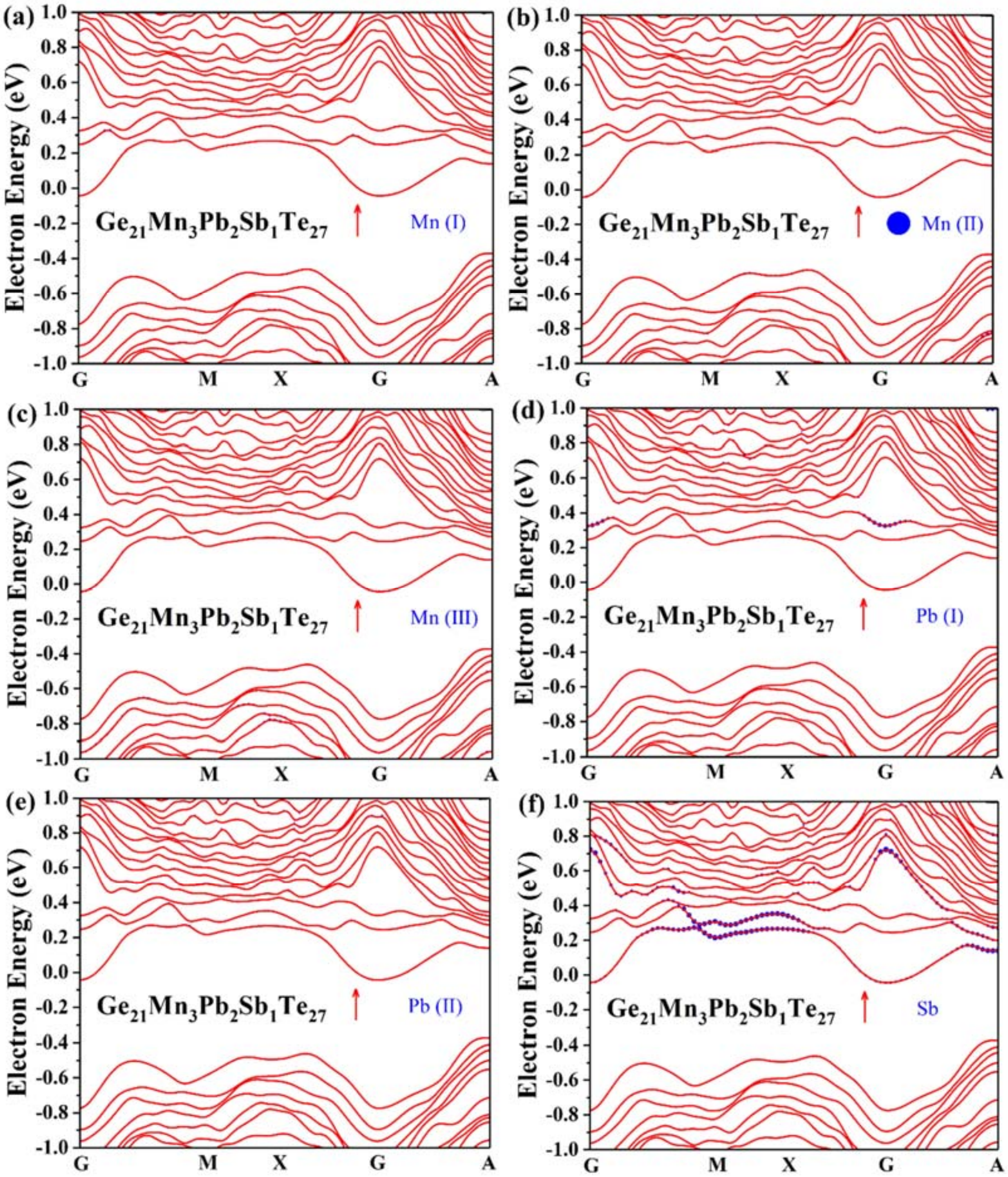

Figure S11. The electronic band structures (spin up) with projected elemental contributions of C-$Ge_{21}Mn_3Pb_2Sb_1Te_{27}$. The Fermi level is positioned at zero energy. The upward red arrow represents the spin up polarized electron band structures.

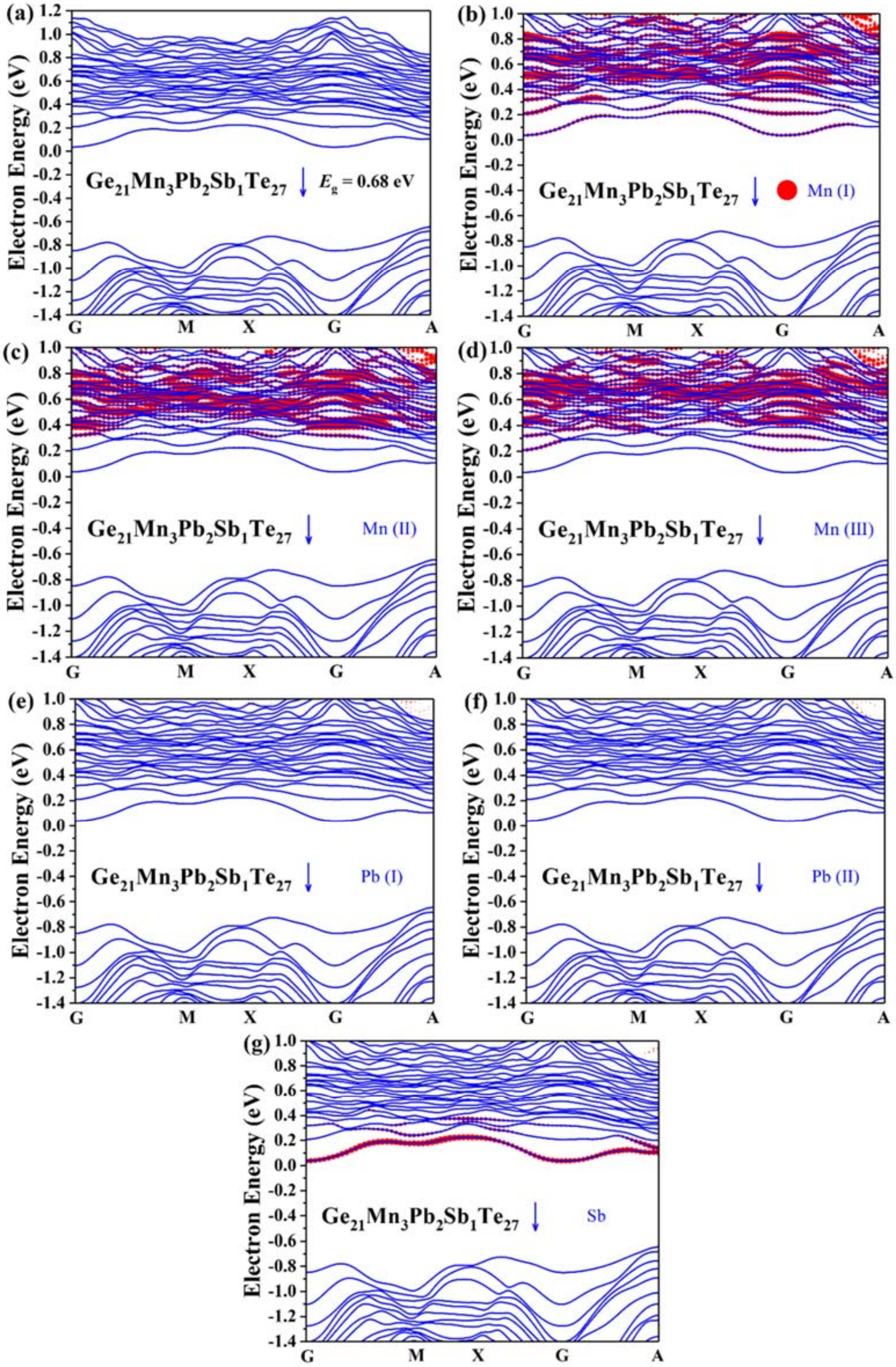

Figure S12. (a) Electron band structures (spin down) of C-$Ge_{21}Mn_3Pb_2Sb_1Te_{27}$. (b)-(g) The electronic band structures (spin down) with projected elemental contributions of C-$Ge_{21}Mn_3Pb_2Sb_1Te_{27}$. The Fermi level is positioned at zero energy. The downward blue arrow represents the spin up polarized electron band structures.

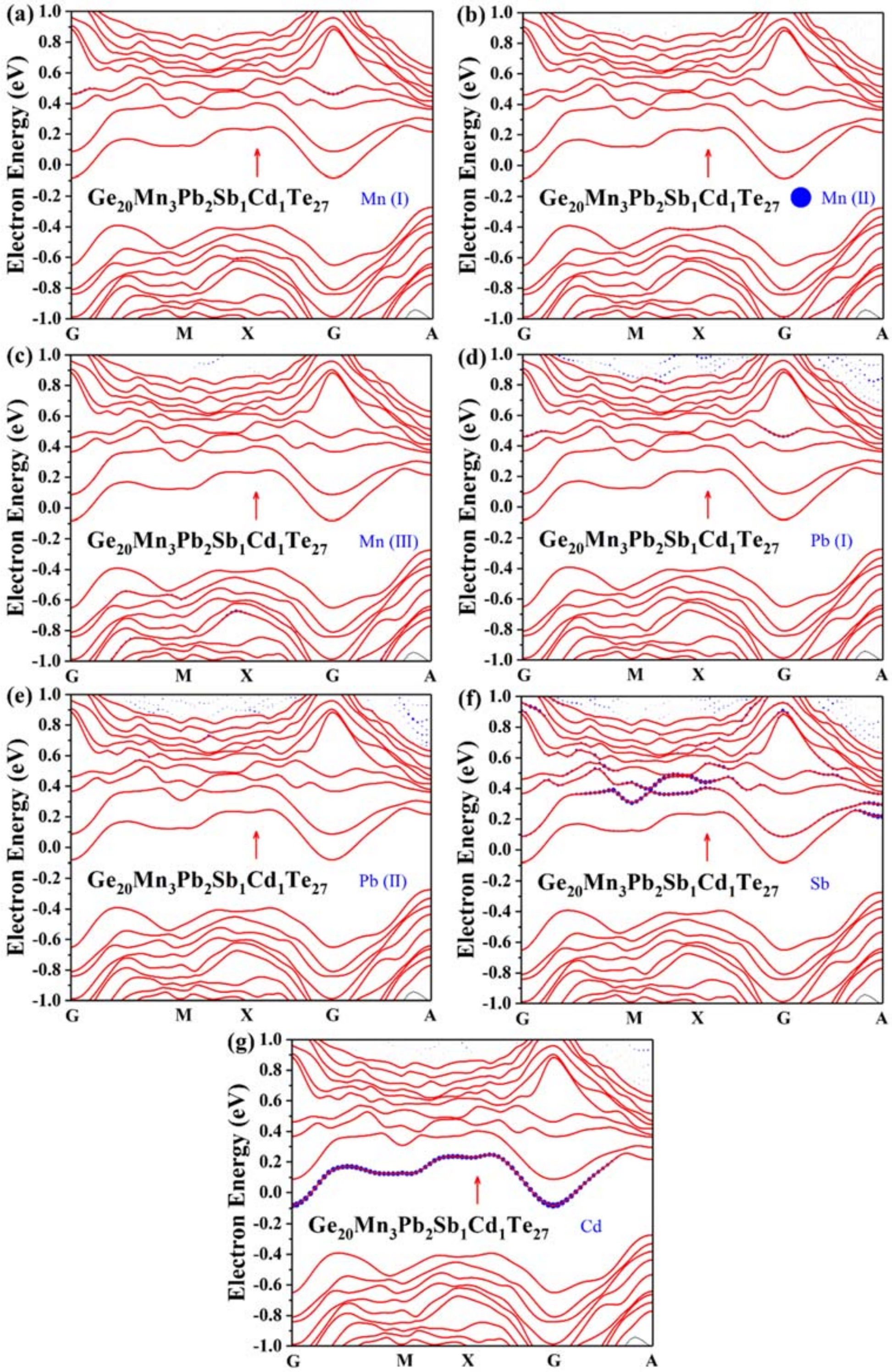

Figure S13. The electronic band structures (spin up) with projected elemental contributions of C-$Ge_{20}Mn_3Pb_2Sb_1Cd_1Te_{27}$. The Fermi level is positioned at zero energy. The upward red arrow represents the spin up polarized electron band structures.

An in-depth examination reveals that the impurity band is the hybridization of dominant Cd with some Sb. By contrast, only Cd contributed to the formation of impurity band in $Ge_{1-x}Cd_xTe$.[9]

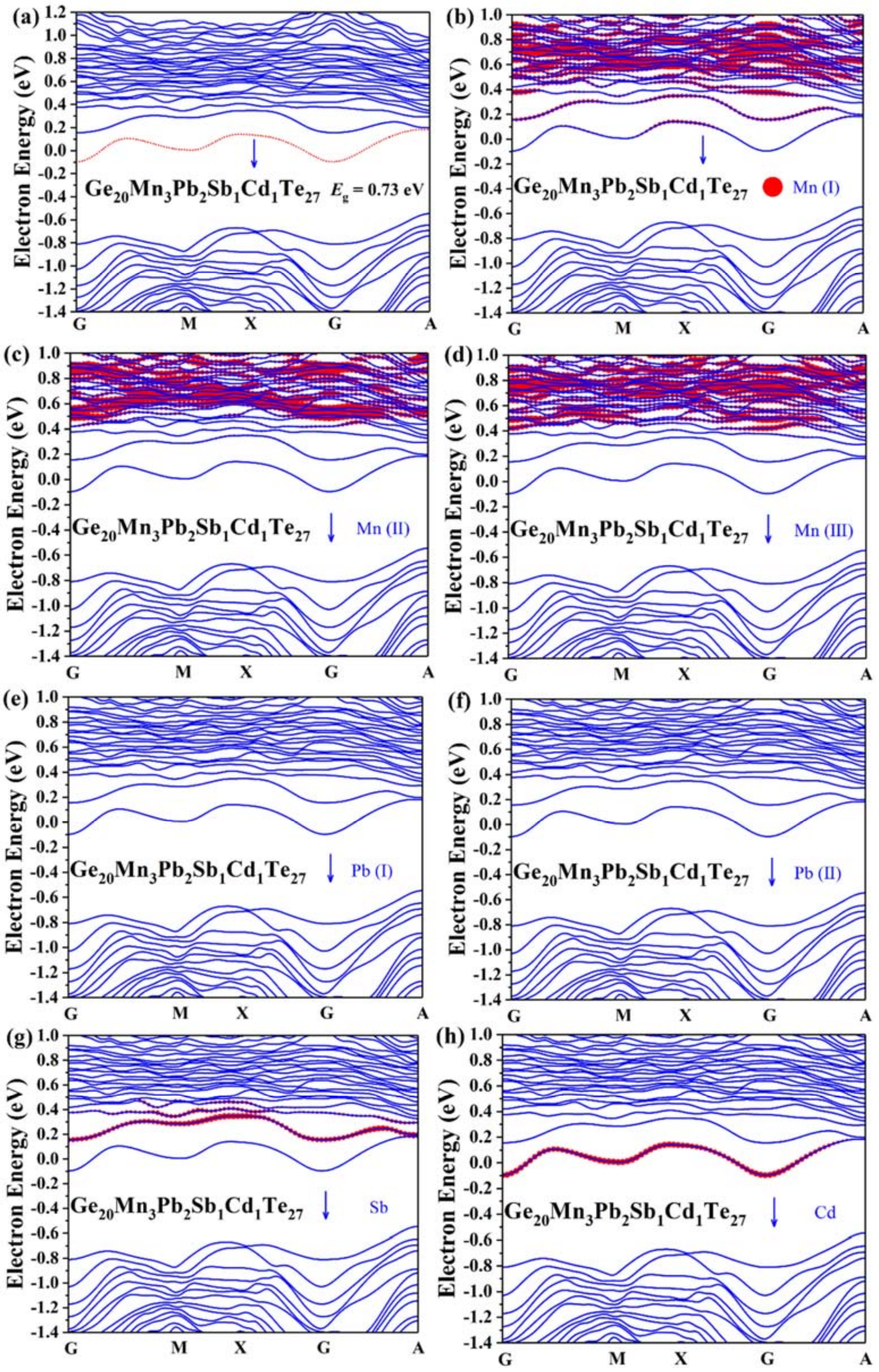

Figure S14. (a) Electron band structures (spin down) of C-$Ge_{20}Mn_3Pb_2Sb_1Cd_1Te_{27}$. (b)-(h) The electronic band structures (spin down) with projected elemental contributions of C-$Ge_{20}Mn_3Pb_2Sb_1Cd_1Te_{27}$. The Fermi level is positioned at zero energy. The downward blue arrow represents the spin up polarized electron band structures.

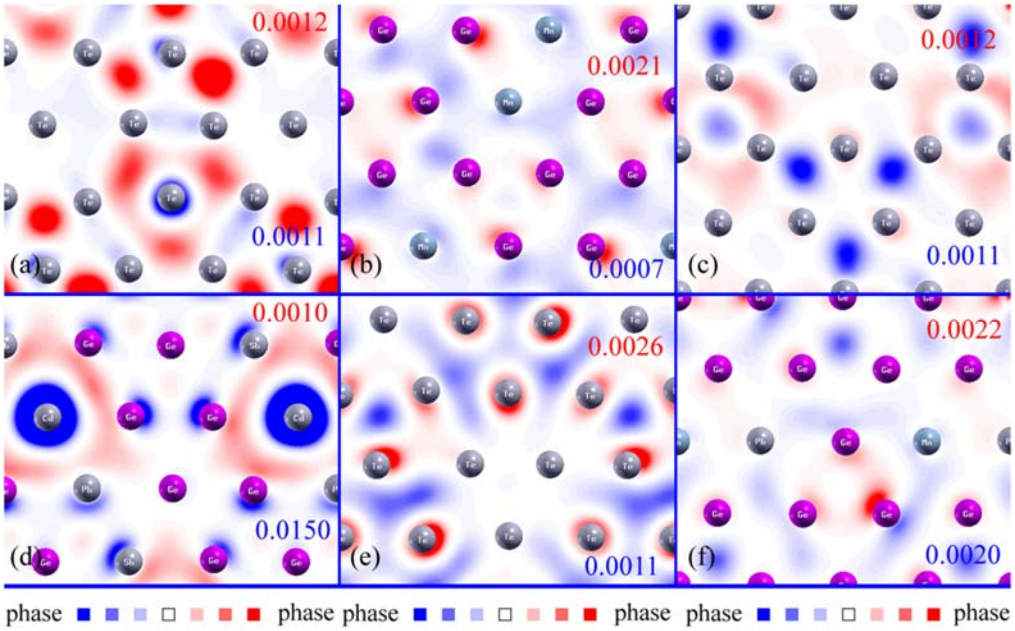

Figure S15. Electron density of the impurity band at gamma point (spin up) of C-$Ge_{20}Mn_3Pb_2Sb_1Cd_1Te_{27}$. (a) to (f) are six successive cutting planes in the unit cell.

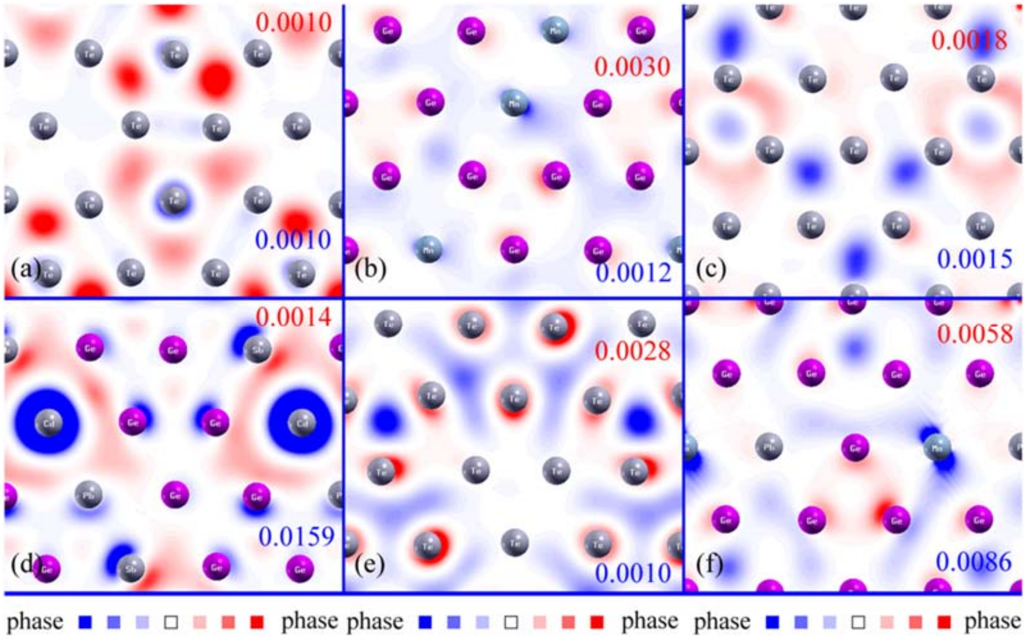

Figure S16. Electron density of the impurity band at gamma point (spin down) of C-$Ge_{20}Mn_3Pb_2Sb_1Cd_1Te_{27}$. (a) to (f) are six successive cutting planes in the unit cell.

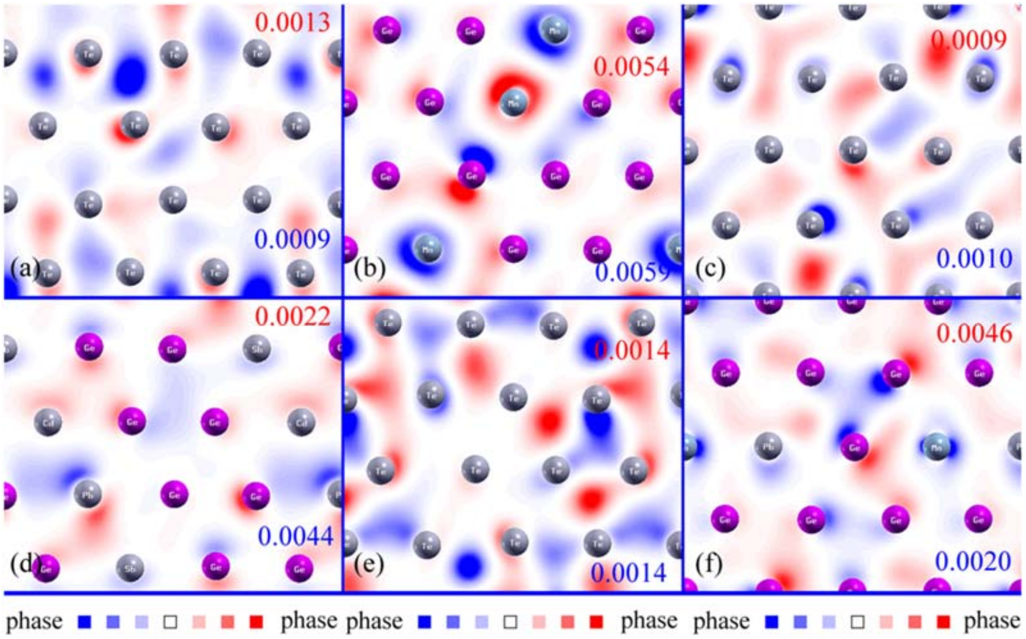

Figure S17. Electron density of conduction band at gamma point (spin up) of C-$Ge_{20}Mn_3Pb_2Sb_1Cd_1Te_{27}$. (a) to (f) are six successive cutting planes in the unit cell.

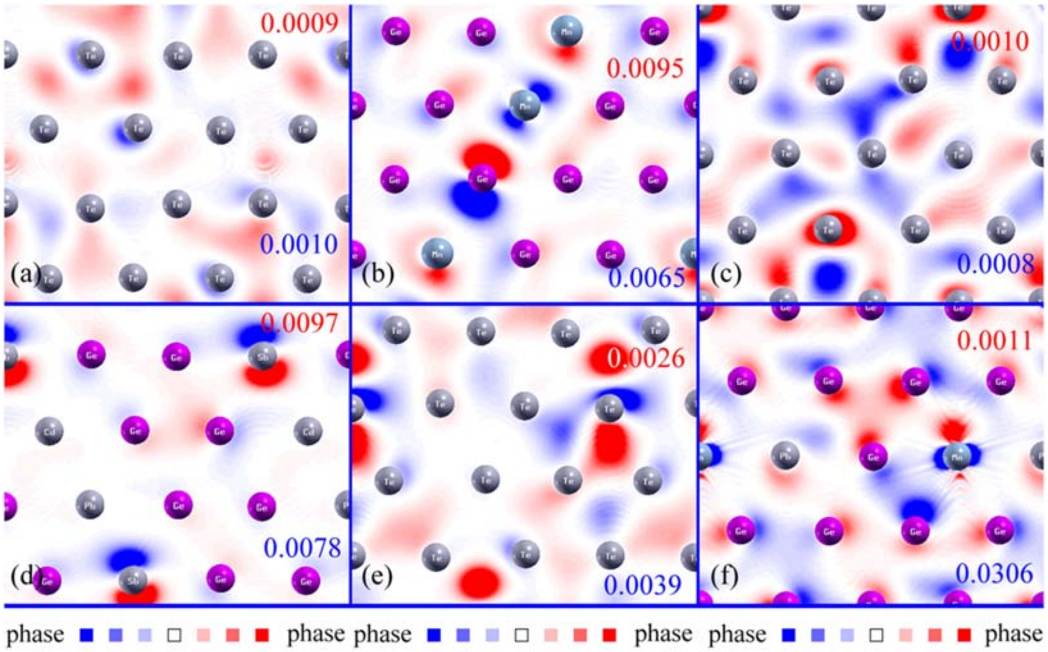

Figure S18. Electron density of conduction band at gamma point (spin down) of C-$Ge_{20}Mn_3Pb_2Sb_1Cd_1Te_{27}$. (a) to (f) are six successive cutting planes in the unit cell.

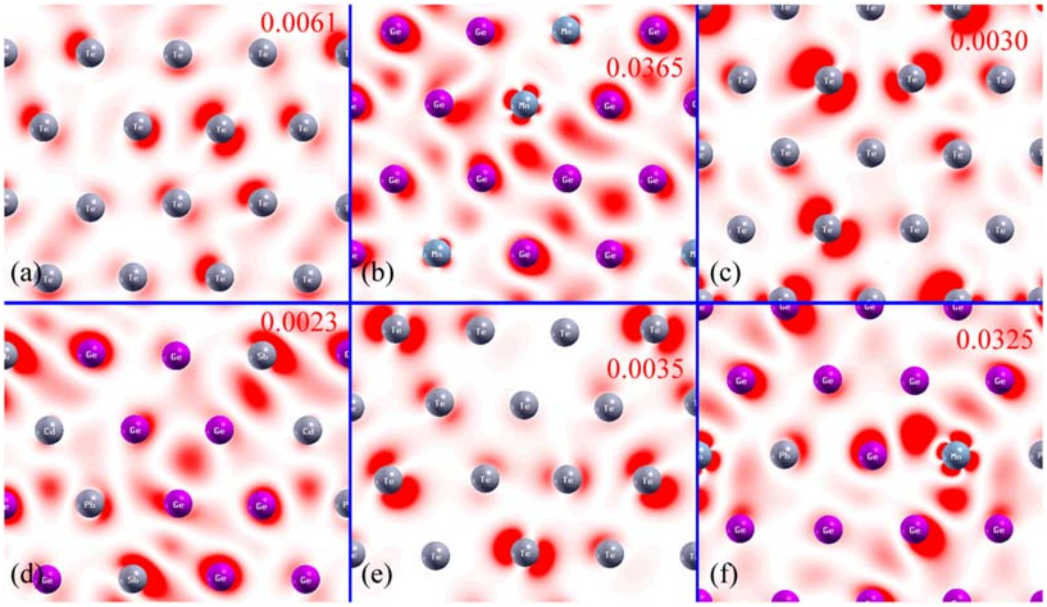

Figure S19. Electron density of valence band at point A (spin up) of C-$Ge_{20}Mn_3Pb_2Sb_1Cd_1Te_{27}$. (a) to (f) are six successive cutting planes in the unit cell.

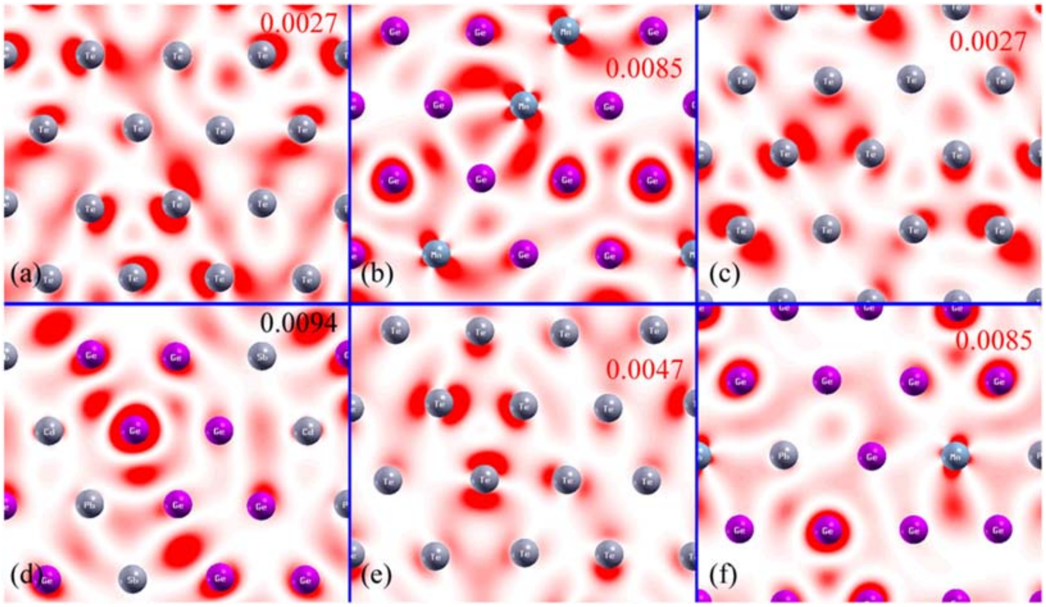

Figure S20. Electron density of valence band at point A (spin down) of C-$Ge_{20}Mn_3Pb_2Sb_1Cd_1Te_{27}$. (a) to (f) are six successive cutting planes in the unit cell.

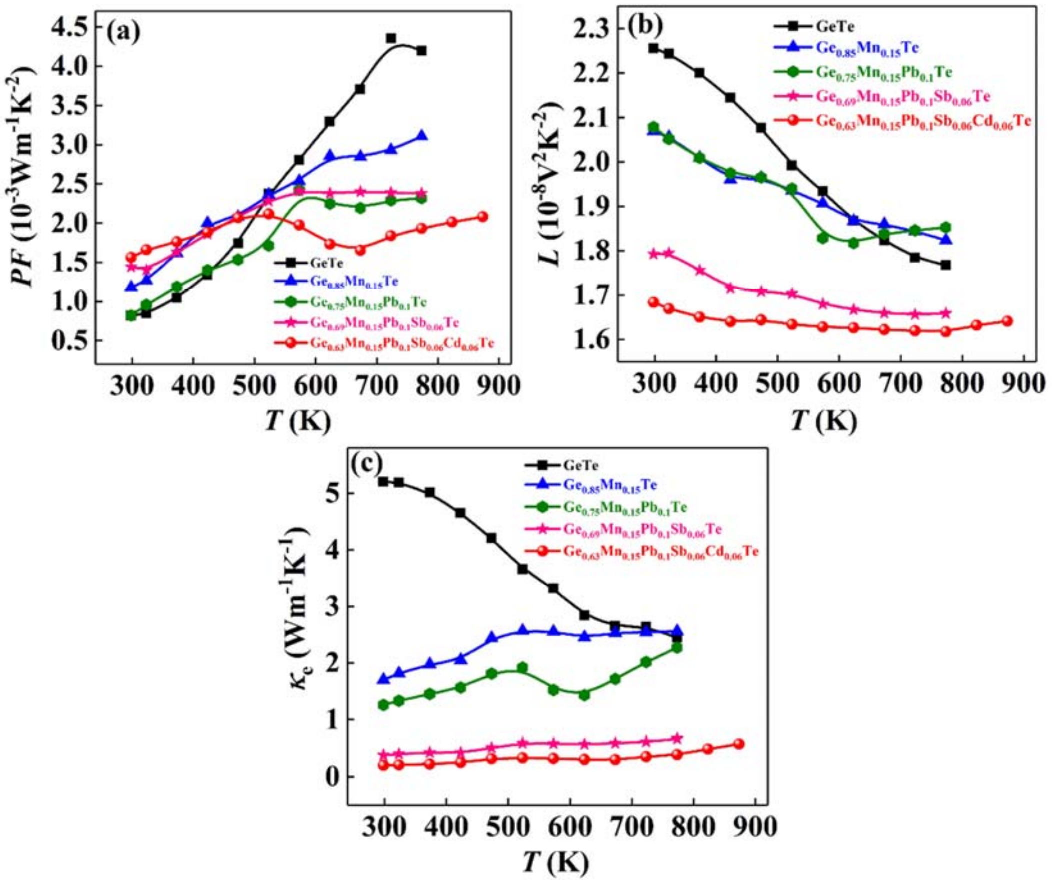

Figure S21. Temperature dependent (a) power factor, (b) Lorenz number, and (c) electrical thermal conductivity of GeTe, $Ge_{0.85}Mn_{0.15}Te$, $Ge_{0.75}Mn_{0.15}Pb_{0.1}Te$, $Ge_{0.69}Mn_{0.15}Pb_{0.1}Sb_{0.06}Te$, and $Ge_{0.63}Mn_{0.15}Pb_{0.1}Sb_{0.06}Cd_{0.06}Te$ samples.

The observed substantial reduction of κ as increasing number of alloy elements is ascribed to the decreased κ_{el} and κ_{ph}. The κ_{el} is estimated by the Wiedemann-Franz law, $\kappa_{el} = L\sigma T$, where the Lorenz number L was calculated using the formula $L = 1.5+\exp(-|S|/116)$.[24]

The downturn in the plot of α versus T (400 – 600 K) at high temperatures for GeTe-based HEAs including $Ge_{0.25}Sn_{0.25}Pb_{0.25}Mn_{0.25}Te$[25] and $Ge_{1/3}Sn_{1/3}Pb_{1/3}Te_{1/3}Se_{1/3}S_{1/3}$[26] is an indicator of the detrimental bipolar effect (Figure 4c). Herein, the wide band gap, impurity band and high n_H in our (Mn,Pb,Sb,Cd) co-alloyed sample effectively mitigates the intrinsic conduction. As signified by the temperature-dependent α (Figure 4c), no obvious contribution from bipolar effect is traced until 773 K.

Similar to the variation of α versus T (Figure 4c), our $Ge_{0.63}Mn_{0.15}Pb_{0.1}Sb_{0.06}Cd_{0.06}Te$ sample has large bandgap, impurity band, and high n_H and thereupon suppressed bipolar effect at high temperature, leading to sustained fall in κ_{ph} with increasing T (Figure 5b). As a result, the distinguished zT ~ 2.1 at 873 K was attained for $Ge_{0.63}Mn_{0.15}Pb_{0.1}Sb_{0.06}Cd_{0.06}Te$ sample due to the suppressed bipolar effect.

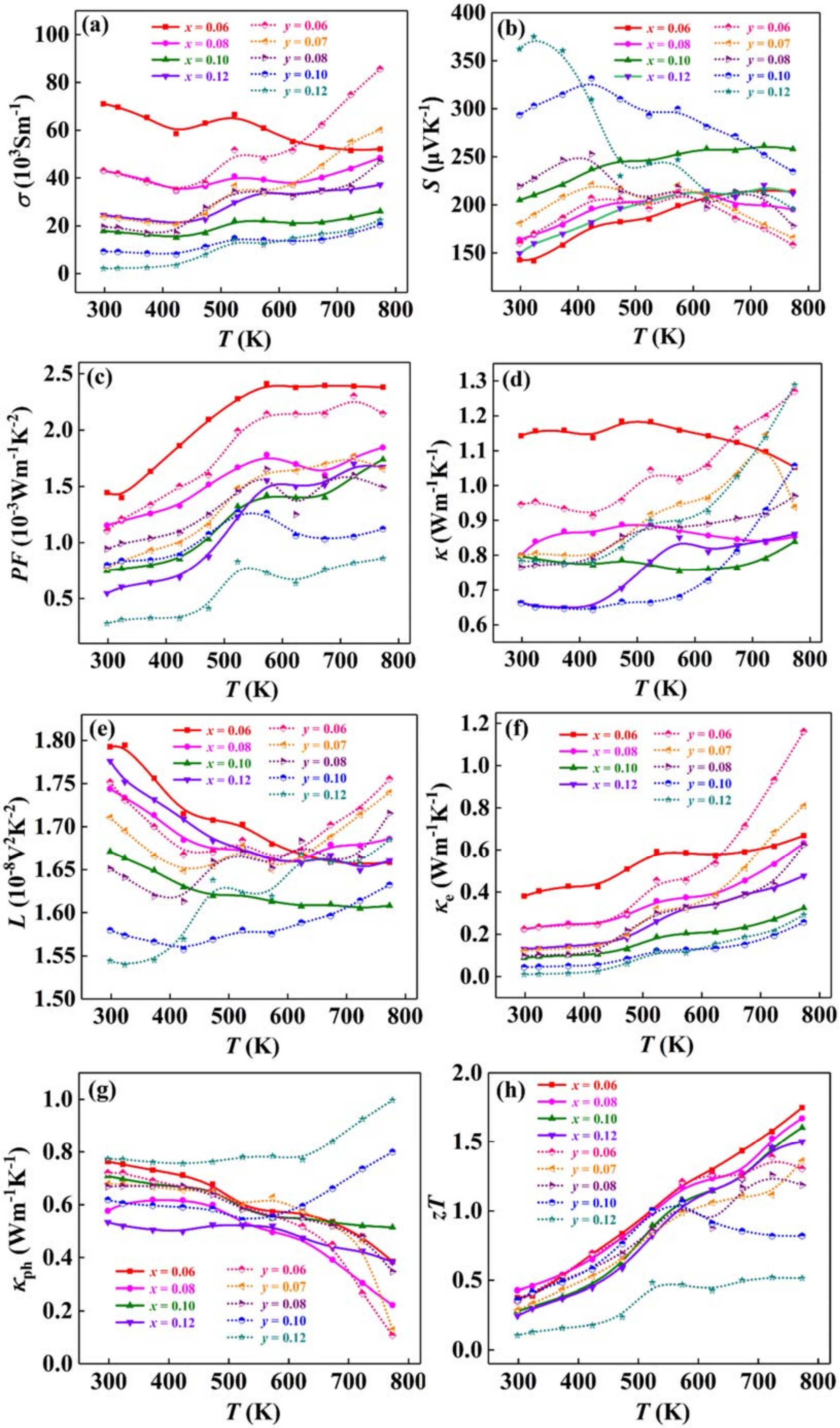
(a)
x = 0.06
x = 0.08
x = 0.10
x = 0.12
y = 0.06
y = 0.07
y = 0.08
y = 0.10
y = 0.12
σ (10³Sm⁻¹)
T (K)
(b)
S (μVK⁻¹)
T (K)
(c)
PF (10⁻³Wm⁻¹K⁻²)
T (K)
(d)
κ (Wm⁻¹K⁻¹)
T (K)
(e)
L (10⁻⁸V²K⁻²)
T (K)
(f)
κe (Wm⁻¹K⁻¹)
T (K)
(g)
κph (Wm⁻¹K⁻¹)
T (K)
(h)
zT
T (K)

Figure S22. Temperature dependent (a) electrical conductivity, (b) Seebeck coefficient, (c) power factor, (d) total thermal conductivity, (e) Lorenz number, (f) electrical thermal conductivity, (g) lattice thermal conductivity, and (f) Figure of Merit *zT* of $Ge_{0.75-x}Mn_{0.15}Pb_{0.1}Sb_xTe$ and $Ge_{0.75-y}Mn_{0.15}Pb_{0.1}Bi_yTe$ series.

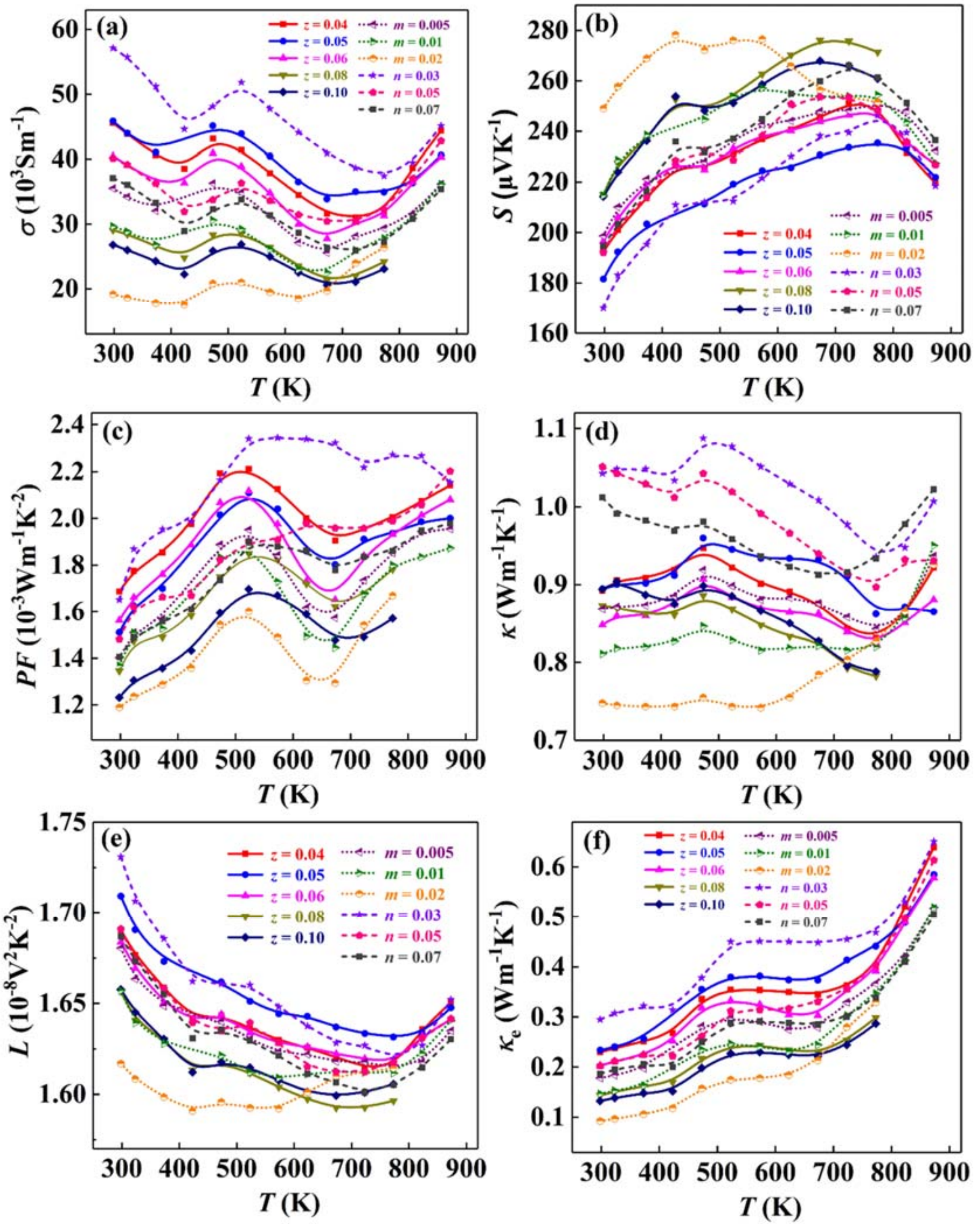
(a)
σ (10^3Sm^-1)
(b)
S (μVK^-1)
(c)
PF (10^-3Wm^-1K^-2)
(d)
κ (Wm^-1K^-1)
(e)
L (10^-8V^2K^-2)
(f)
κe (Wm^-1K^-1)
T (K)
z = 0.04
z = 0.05
z = 0.06
z = 0.08
z = 0.10
m = 0.005
m = 0.01
m = 0.02
n = 0.03
n = 0.05
n = 0.07

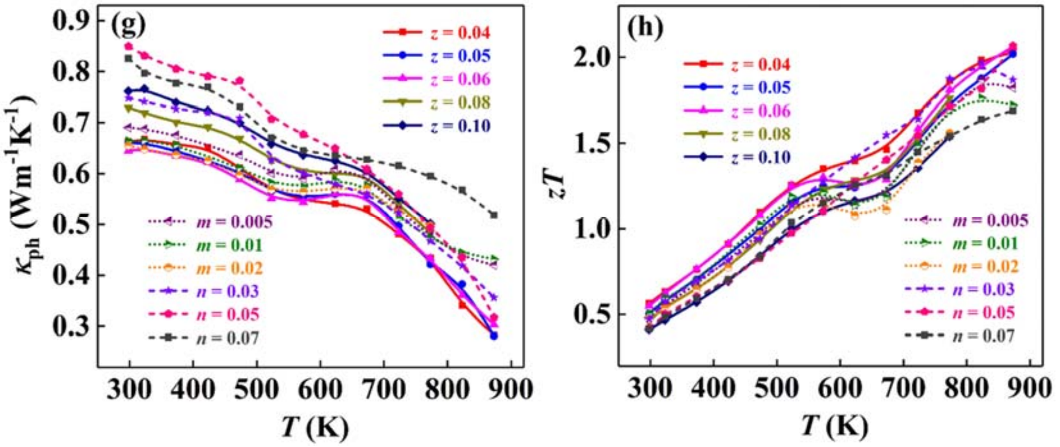

Figure S23. Temperature dependent (a) electrical conductivity, (b) Seebeck coefficient, (c) power factor, (d) total thermal conductivity, (e) Lorenz number, (f) electrical thermal conductivity, (g) lattice thermal conductivity, and (f) Figure of Merit zT of $Ge_{0.69-z}Mn_{0.15}Pb_{0.1}Sb_{0.06}Cd_zTe$, $Ge_{0.64-m}Mn_{0.15}Pb_{0.1}Sb_{0.06}Cd_{0.05}In_mTe$, and $Ge_{0.64-n}Mn_{0.15}Pb_{0.1}Sb_{0.06}Cd_{0.05}Zn_nTe$ series.

We in supplemental studies have tried six alloy elements, including $Ge_{0.64-m}Mn_{0.15}Pb_{0.1}Sb_{0.06}Cd_{0.05}In_mTe$, and $Ge_{0.64-n}Mn_{0.15}Pb_{0.1}Sb_{0.06}Cd_{0.05}Zn_nTe$ series, as shown in Figure S23, Supporting Information.

On one hand, the gradual saturation of phonon scattering from atomic disorder through atomic mass and strain fluctuations has been observed as the number of components increases from one to five (Figure 5c), limiting the persistent reduction in κ_{ph}. As depicted in Figure S23, Supporting Information, alloying six elements, i.e. In or Zn alloying in this work, cannot induce the further reduction in κ_{ph}. This confirms that medium-entropy alloying is sufficient to suppress the κ_{ph} to the glass limit of a solid for GeTe with moderate initial κ_{ph}.

On the other hand, despite the α has been enhanced upon further In or Zn alloying, the continuous degradation of μ_H and PF excludes any thermoelectric promise by alloying more elements. However, we do not consider these observations "general", as they may depend on "what to alloy", "how much to alloy" and maybe other hidden parameters.

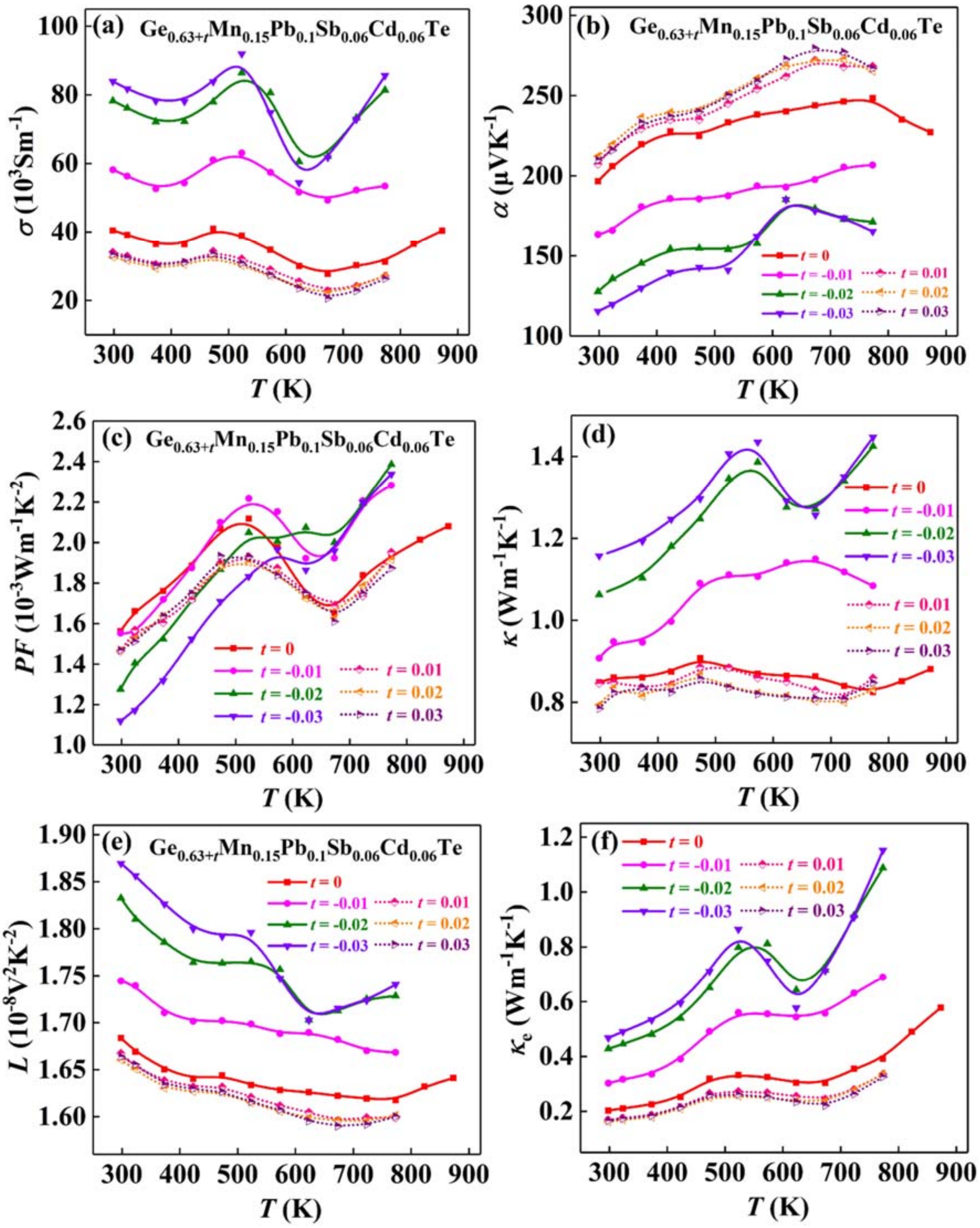
(a) $Ge_{0.63+t}Mn_{0.15}Pb_{0.1}Sb_{0.06}Cd_{0.06}Te$
σ (10^3 Sm^-1)
T (K)
(b) $Ge_{0.63+t}Mn_{0.15}Pb_{0.1}Sb_{0.06}Cd_{0.06}Te$
α (μVK^-1)
(c) $Ge_{0.63+t}Mn_{0.15}Pb_{0.1}Sb_{0.06}Cd_{0.06}Te$
PF (10^-3 Wm^-1K^-2)
(d)
κ (Wm^-1K^-1)
(e) $Ge_{0.63+t}Mn_{0.15}Pb_{0.1}Sb_{0.06}Cd_{0.06}Te$
L (10^-8 V^2K^-2)
(f)
κe (Wm^-1K^-1)
t = 0
t = -0.01
t = -0.02
t = -0.03
t = 0.01
t = 0.02
t = 0.03

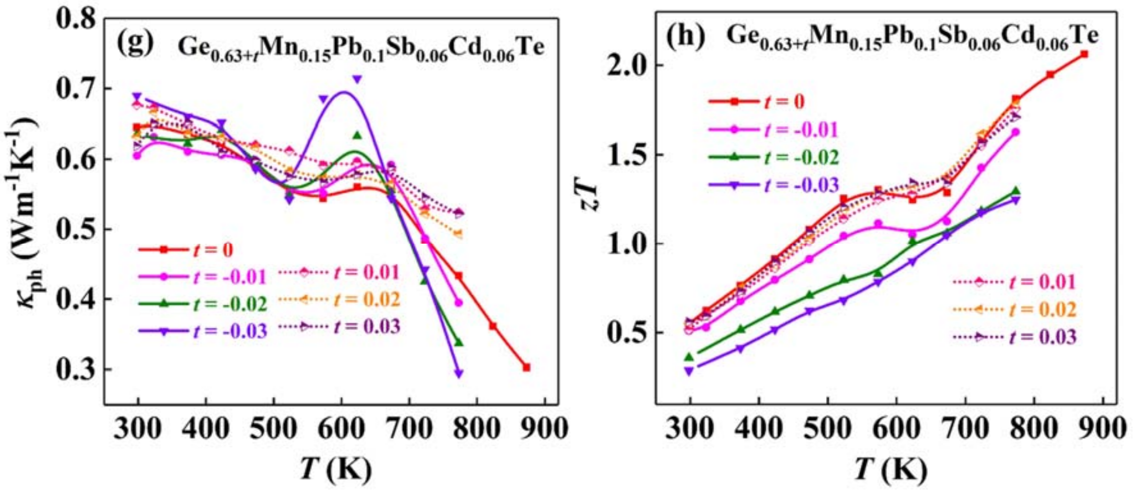

Figure S24. Temperature dependent (a) electrical conductivity, (b) Seebeck coefficient, (c) power factor, (d) total thermal conductivity, (e) Lorenz number, (f) electrical thermal conductivity, (g) lattice thermal conductivity, and (f) Figure of Merit *zT* of $Ge_{0.63+t}Mn_{0.15}Pb_{0.1}Sb_{0.06}Cd_{0.06}Te$.

We further tune the n_H by Ge self-doping in the $Ge_{0.63+t}Mn_{0.15}Pb_{0.1}Sb_{0.06}Cd_{0.06}Te$ (t = -0.03, -0.02, -0.01, 0.01, 0.02, and 0.03) series, as shown in Figure S24, Supporting Information. As can be seen, the decreased σ and increased α with increasing Ge excess (i.e. t = 0.01 - 0.03) indicates a reduced n_H. As a result, the slightly decreased *PF* because of the reduced σ and the increased κ_{ph} owing to the diminished concentration of Ge vacancies both limit the further improvement of *zT*. Nevertheless, the similar high *zT* values of 1.71 - 1.78 at 773 K are attained for these Ge excess samples compared to $Ge_{0.63}Mn_{0.15}Pb_{0.1}Sb_{0.06}Cd_{0.06}Te$ sample (*zT* ~ 1.81 at 773 K), attesting the repeatability of present work.

On the contrary, the n_H and hence the σ is greatly increased by simply increasing the amount of Ge short (i.e. t = -0.01, -0.02, and -0.03). At the same time, Ge vacancies enhance phonon scattering and substantially decrease the κ_{ph}. Unfortunately, the remarkably increased κ_{el} and drastically reduced α due to the increased n_H leads to a drastically degraded *zT*. These results indicate that the fully optimized n_H is indeed achievable in the present work.

Supporting References

[1] Y.Zheng, Q. Zhang, X. L. Su, H. Y. Xie, S. C. Shu, T. Chen, G. J. Tan, Y. G. Yan, X. F. Tang, C. Uher, G. J. Snyder, *Adv. Energy Mater*. **2015**, *5*, 1401391.

[2] A. C. Larson, R. B. Von Dreele, General structure analysis system (GSAS), Report No. LAUR 86-748, Los Alamos National Labortory, Los Alamos, NM, 2000.

[3] B. H. Toby, *J. Appl. Crystallorgr*. **2001**, *34*, 210.

[4] F. Fahrnbauer, D. Souchay, G. Wagner, O. Oeckler, *J. Am. Chem. Soc*. **2015**, *139*, 12633.

[5] J. Li, Z. W. Chen, X. Y. Zhang, Y. X. Sun, J. Yang, Y. Z. Pei, *NPG Asia Mater*. **2017**, *9*, e353.

[6] S. Perumal, P. Bellare, U. S. Shenoy, U. V. Waghmare, K. Biswas, *Chem. Mater*. **2017**, *29*, 10426−10435.

[7] J. Li, X. Y. Zhang, Z. W. Chen, S. Q. Lin, W Li, J. H. Shen, I. T. Witting, A Faghaninia, Y. Chen, A Jain, L. D. Chen, G. J. Snyder, Y. Z. Pei, *Joule* **2018**, *2*, 976-987.

[8] J. Li, X. Y. Zhang, X. Wang, Z. L. Bu, L. T. Zheng, B. Q. Zhou, F. Xiong, Y. Chen, Y. Z. Pei, *J. Am. Chem. Soc*. **2018**, *140*, 16190.

[9] M. Hong, Z. G. Chen, L. Yang, Y. C. Zou, M. S. Dargusch, H. Wang, J. Zou, *Adv. Mater*. **2018**, *30*, 1705942.

[10] M. Hong, K. Zheng, W. Y. Lyv, M. Li, X. L. Qu, Q. Sun, S. D. Xu, J. Zou, Z. G. Chen, *Energy Environ. Sci*. **2020**, *13*, 1856.

[11] M. Hong, Y. Wang, T. L. Feng, Q. Sun, S. D. Xu, S. Matsumura, S. T. Pantelides, J. Zou, Z. G. Chen, *J. Am. Chem. Soc*. **2019**, *141*, 1742.

[12] Z. Zheng, X. L. Su, R. G. Deng, C. Stoumpos, H. Y. Xie, W. Liu, Y. G. Yan, S. Q. Hao, C. Uher, C. Wolverton, M. G. Kanatzidis, X. F. Tang, J. *Am. Chem. Soc*. **2018**, *140*, 2673.

[13] M. Samanta, K. Biswas, *J. Am. Chem. Soc*. **2017**, *139*, 9382.

[14] Y. Gelbstein, J. Davidow, S. N. Girard, D. Y. Chung, M. Kanatzidis, *Adv. Energy Mater*. **2013**, *3*, 815.

[15] S. Welzmiller, F. Fahrnbauer, F. Hennersdorf, S. Dittmann, M. L, C. Fraunhofer, W. G. Zeier, G. J. Snyder, O. Oeckler, *Adv. Electron. Mater*. **2015**, *1*, 1500266.

[16] Y. Gelbstein, J. Davidow, *Phys. Chem. Chem. Phys.* **2014**, *16,* 20120.

[17] Z. Zheng, X. L. Su, R. G. Deng, C. Stoumpos, H. Y. Xie, W. Liu, Y. G. Yan, S. Q. Hao, C. Uher, C. Wolverton, M. G. Kanatzidis, X. F. Tang, J. *Am. Chem. Soc*. **2018**, *140*, 2673.

[18] J. Li, X. Y. Zhang, X. Wang, Z. L. Bu, L. T. Zheng, B. Q. Zhou, F. Xiong, Y. Chen, Y. Z. Pei, *J. Am. Chem. Soc*. **2018**, *140*, 16190.

[19] S. Perumal, S. Roychowdhury, D. S. Negi, R. Datta, K. Biswas, *Chem. Mater*. **2015**, *27*, 7171−7178.

[20] J. Li, W. Li, Z. L. Bu, X. Wang, B. Gao, F. Xiong, Y. Chen, Y. Z. Pei, *ACS Appl. Mater. Interfaces* **2018**, *10*, 39904.

[21] L. P. Hu, Y. Zhang, H. J. Wu, J. Q. Li, Y. Li, M. Mckenna, J. He, F. S. Liu, S. J. Pennycook, X. R. Zeng, *Adv. Energy Mater.* **2018**, *8*, 1802116.

[22] Y. Jin, Y. Xiao, D. Y. Wang, Z. W. Huang, Y. T. Qiu, L. D. Zhao, *ACS Appl. Energy Mater*. **2019**, *2*, 7594.

[23] Y. Q. Cheng, E. Ma, *Prog. Mater. Sci*. **2011**, *56*, 379.

[24] H. S. Kim, Z. M. Gibbs, Y. L. Tang, H. Wang, G. J. Snyder, *APL Mater*. **2015**, *3*, 041506.
